# AHSA1-HSP90AA1 complex stabilized IFI6 and TGFB1 promotes mitochondrial stability and EMT in EGFR-mutated lung adenocarcinoma under Osimertinib pressure

**DOI:** 10.1038/s41419-025-07650-9

**Published:** 2025-04-15

**Authors:** Ying Sui, Ziyang Shen, Rongtian Pan, Rong Ma, Rujia Si, Jifeng Feng, Guoren Zhou

**Affiliations:** 1 The Affiliated Cancer Hospital of Nanjing Medical University, Nanjing Medical University, Nanjing, Jiangsu China; 2 Jiangsu Cancer Hospital, and Jiangsu Institute of Cancer Research, Nanjing, Jiangsu China

**Keywords:** Apoptosis, Non-small-cell lung cancer

## Abstract

Tyrosine kinase inhibitors (TKIs) have substantially improved the management of lung adenocarcinoma harboring epidermal growth factor receptor (EGFR) mutations, however, not all patients can derive benefit from it. We found that the overexpression of IFI6 under the influence of the AHSA1-HSP90AA1 complex significantly enhances Osimertinib resistance in EGFR-mutated lung adenocarcinoma cells. This effect is achieved by stabilizing mitochondrial function, reducing apoptosis, and promoting cell survival pathways via increased Akt phosphorylation. Additionally, we revealed that TGFB1 further promotes epithelial-mesenchymal transition (EMT) and enhances the invasive and migratory capabilities of these cells, thereby intensifying resistance. Regarding mechanisms, the AHSA1-HSP90AA1 complex stabilizes IFI6 and TGFB1 to enhance cell survival and Osimertinib resistance in EGFR mutant lung adenocarcinoma. IFI6 not only aids in cellular survival under drug stress but also promotes aggressive tumor phenotypes, suggesting its viability as a novel biomarker and therapeutic target for overcoming primary TKI resistance.

## Introduction

Tyrosine kinase inhibitors (TKIs) have significantly transformed the management and prognosis of patients with lung adenocarcinoma (LUAD) harboring epidermal growth factor receptor (EGFR) sensitizing mutations [1–3]. Although patients treated with EGFR-TKIs experience extended progression-free survival and high tumor response rates, On-target and off-target resistance pose significant obstacles to this therapy[4, 5]. Cell stress factors and their downstream activation play an indispensable role in the resistance to EGFR-TKIs [6, 7]. Whether it is the downstream IL-6 signaling activation caused by LKB1 inhibition under TKI pressure, or TKI resistance induced by secondary hypoxic stress, the impact of these TKI resistance-related molecules and mechanisms on prognosis and predictive value is increasingly being elucidated [8, 9].

AHSA1 (Activator of HSP90AA1 ATPase Activity 1) activates the ATPase activity of HSP90AA1, thereby enhancing its function. HSP90AA1, a cellular stress response protein, is expressed more highly in cancerous tissues than in normal tissues and has been targeted by anticancer therapies for decades [10–14]. Numerous clinical trials involving HSP90AA1 inhibitors have emerged; however, most have not become standard treatments due to the adverse side effects and toxicity that patients find difficult to tolerate [15, 16]. This is largely because these inhibitors are too broad-spectrum, suppressing many downstream molecules bound to HSP90AA1 in addition to HSP90AA1 itself [17–19]. Targeting more specific downstream targets of HSP90AA1 may provide a more viable approach.

In this study, we have revealed that IFI6, an apoptosis-regulating gene, serves as a direct downstream component of the AHSA1/HSP90AA1 complex. It stabilizes mitochondrial function in EGFR mutant LUAD cells and aids in resistance to Osimertinib stress. This insight promises to provide a new theoretical basis and clinical reference for overcoming primary off-target Osimertinib resistance.

## Results

### AHSA1 alleviates the Osimertinib-induced stress in EGFR-mutated lung adenocarcinoma

**Fig. 1:**
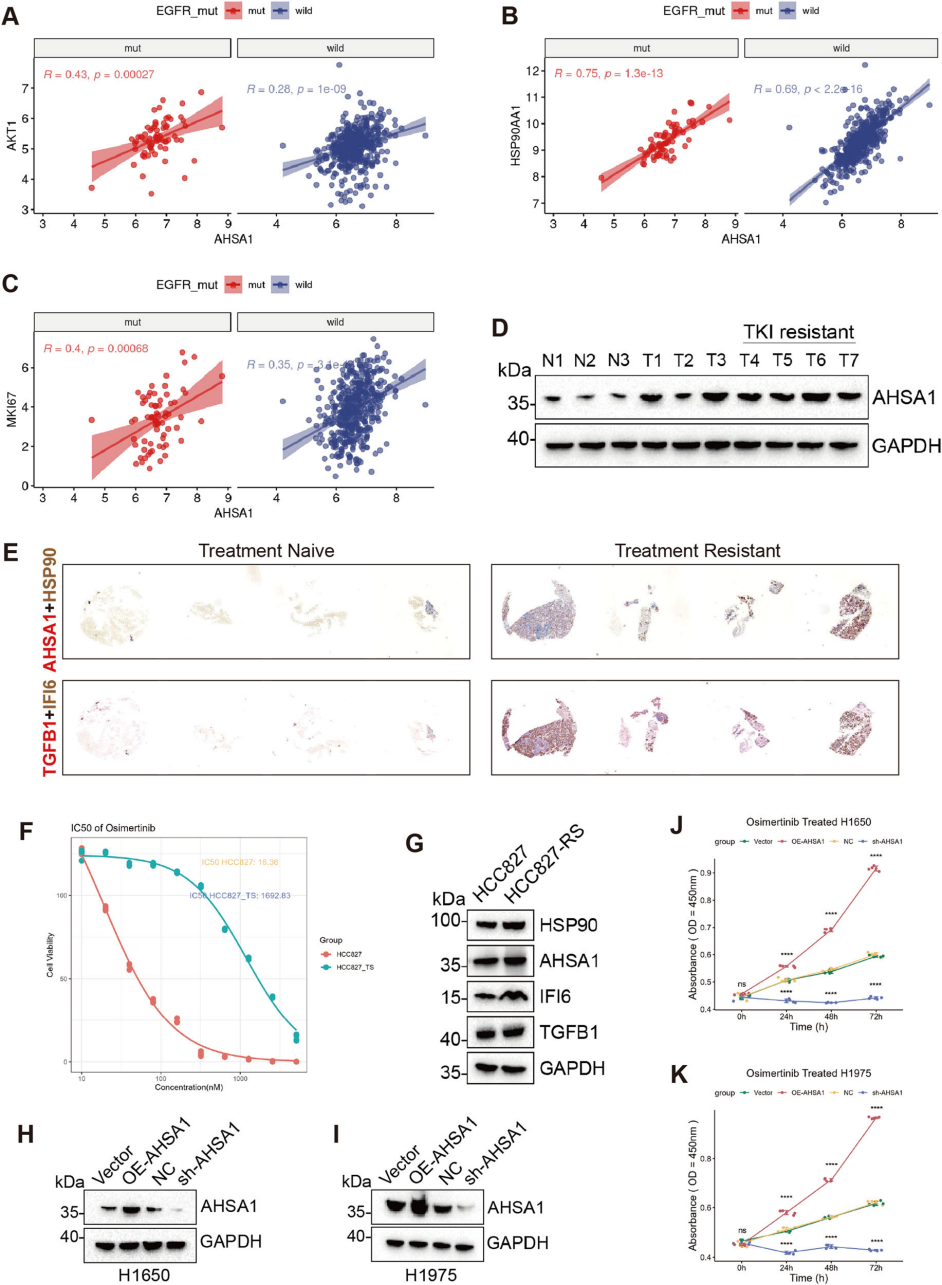
AHSA1 correlates with HSP90AA1 and EGFR-TKI stress in EGFR-mutated lung adenocarcinoma. **A**–**C** Correlation in the gene expression of AHSA1 with AKT1, HSP90AA1, and MKI67 in TCGA-LUAD samples with or without EGFR mutations. **D** The expression of AHSA1 in EGFR mutant LUAD patients was assessed by Western blot (WB) in normal tissue adjacent to the tumor, tumor tissue, and tumor tissue resistant to TKI treatment. **E** The expression of AHSA1, HSP90AA1, IFI6, and TGFB1 in tissues of TKI-sensitive and resistant patients was assessed by immunohistochemistry (IHC). **F** IC50 of HCC827 parental and Osimertinib resistant (HCC827-TS) cells. **G** WB was conducted to assess the expression of AHSA1, HSP90AA1, IFI6, and TGFB1 in HCC827 and HCC827-TS cells. In stably transfected cell lines, **H**, **I** AHSA1 expression was also detected by WB. **J**, **K** Cell viability in these stably transfected cell lines under Osimertinib stress was measured using the CCK-8 assay. Note: *n* = 3, Error bars represent the mean ± SD. *P < 0.05, **P < 0.01, ***P < 0.001, ****P < 0.0001.

AHSA1 is differentially expressed in the TCGA-LUAD cancer versus adjacent non-cancerous samples, and higher expression levels of AHSA1 are correlated with poorer prognosis and increased metastasis (Supplementary Fig. 1A–C). Gene correlation analysis revealed that compared to samples with wild-type EGFR, AHSA1 in EGFR mutant samples exhibits stronger positive correlations with HSP90AA1, AKT1, and MKI67(Fig. 1A–C). Given previous studies reporting that AHSA1 can bind to HSP90AA1 and resist pharmacological stress, we endeavored to further explore the role of AHSA1 in EGFR mutant LUAD. Western blot (WB) results indicated that in patients with EGFR mutations, AHSA1 expression was higher in biopsied tissues(pre-treatment) compared to those with wild-type EGFR, and even higher in the tissues of patients who are unresponsive to subsequent TKI therapies (Fig. 1D), suggesting that AHSA1 may play a role in the response of EGFR mutant LUAD cells under Osimertinib-induced stress. Immunohistochemistry (IHC) analysis of patient-derived tissue microarrays (pre-treatment samples) revealed elevated expression of AHSA1, HSP90AA1, IFI6, and TGFB1 in TKI-resistant EGFR-mutant LUAD (Fig. 1E). Compared to their parental cells, Osimertinib-resistant HCC827-derived cell lines also exhibited increased expression of AHSA1, HSP90AA1, IFI6, and TGFB1, suggesting a close association between these molecules and Osimertinib resistance (Fig. 1F, G). We developed cell lines overexpressing and knocking down AHSA1 in EGFR mutant contexts (Fig. 1H, I). Proliferation assays showed that the inhibition of EGFR mutant LUAD cells by Osimertinib was significantly pronounced in cells with knocked-down AHSA1(Fig. 1J, K, Supplementary Fig. 1D, E). However, cell lines overexpressing AHSA1 maintained a relatively good proliferation rate under Osimertinib stress, indicating that AHSA1 enhances the resistance of EGFR mutant cells to Osimertinib and supports their proliferation.

### AHSA1 promotes epithelial-mesenchymal transition (EMT) and facilitates cell cycle progression to counteract apoptosis

**Fig. 2:**
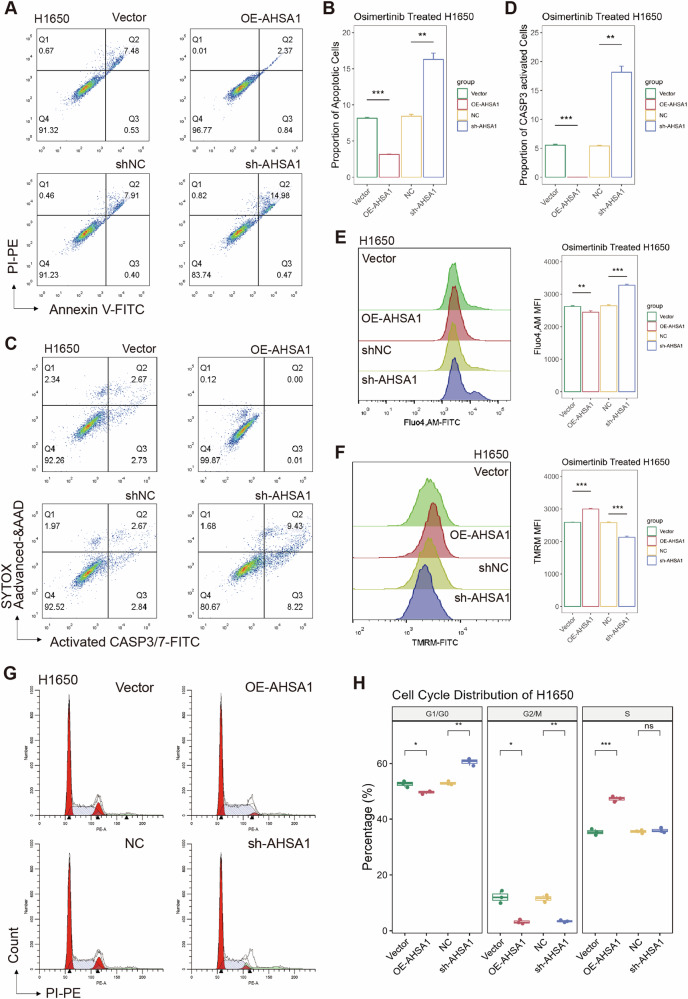
AHSA1 promotes apoptosis evasion, mitochondrial stability, and cell cycle in EGFR-mutated lung adenocarcinoma cells. **A**–**D** Under Osimertinib stress, the apoptosis and CASP3/7 activation status in stably transfected cell lines were assessed. **E** The concentration of intracellular free calcium ions in these cell lines under Osimertinib stress was measured using Fluo-4, AM probe. **F** Mitochondrial activity was examined using TMRM (Tetramethylrhodamine, methyl ester) staining. **G**, **H** The distribution of cell cycle phases in the stably transfected cell lines was analyzed using PI (propidium iodide) staining. Note: *n* = 3, error bars represent the mean ± SD. *P < 0.05, **P < 0.01, ***P < 0.001, ****P < 0.0001.

**Fig. 3:**
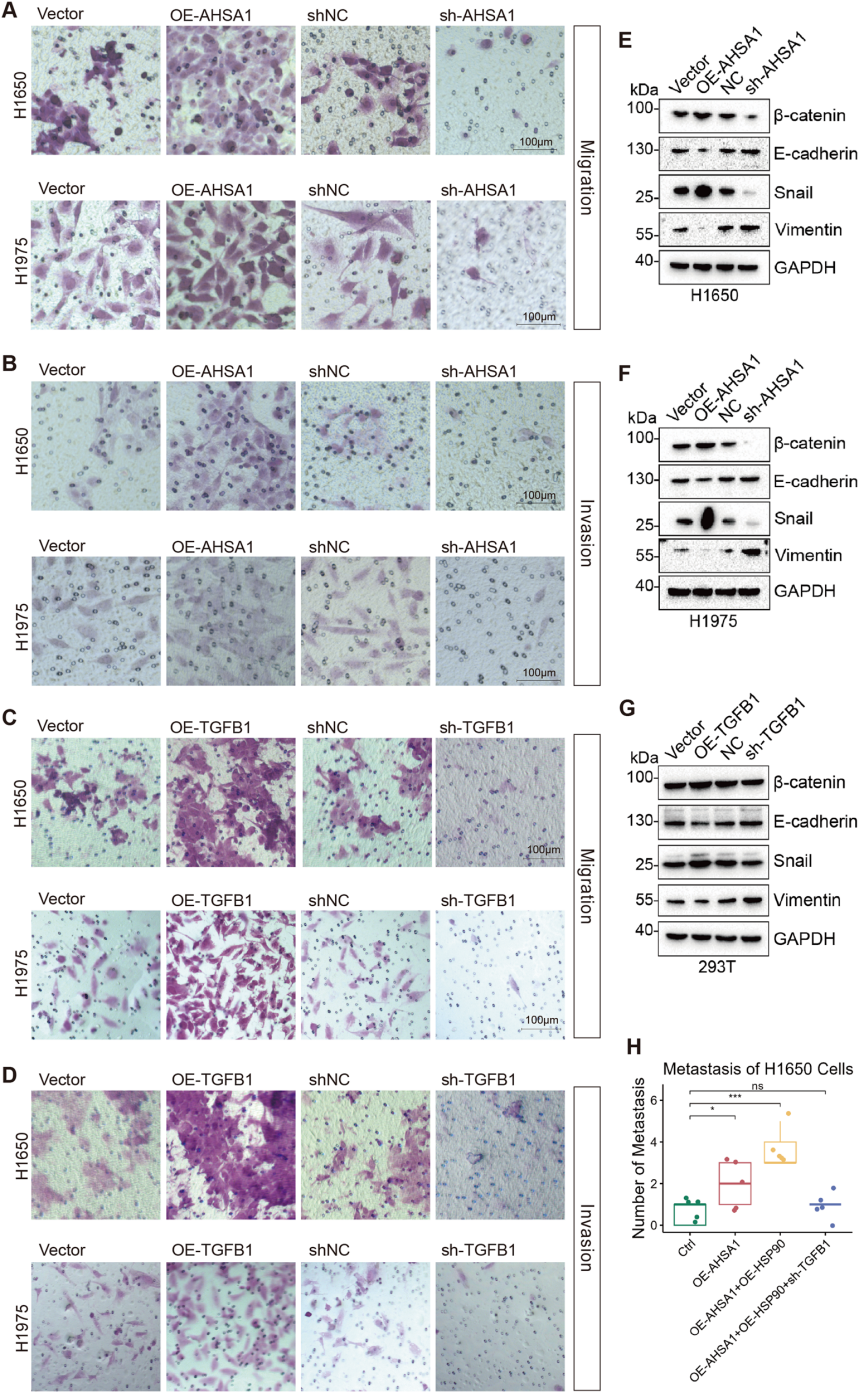
AHSA1 promotes EMT to enhance migration and invasion of EGFR-mutated lung adenocarcinoma cells. **A**, **B** The migration capability of stably transfected cell lines was evaluated using Transwell assays. **C**, **D** The invasion capability of stably transfected cell lines was evaluated using Transwell assays. **E**–**G** The expression levels of β-catenin, E-cadherin, Snail, and Vimentin in cell lines were assessed by WB. **H** The number of metastatic lesions in the tail vein lung metastasis model was quantified. Note: *n* = 3, error bars represent the mean ± SD. *P < 0.05, **P < 0.01, ***P < 0.001, ****P < 0.0001.

Apoptosis assays demonstrated that overexpressing AHSA1 reduced apoptosis in EGFR mutant LUAD cells under Osimertinib stress and inhibited CASP3/7 activation (Fig. 2A–D, Supplementary Fig. 1F–I); conversely, knocking down AHSA1 rendered EGFR mutant LUAD cells more sensitive to Osimertinib, with an increased proportion of cells exhibiting CASP3/7 activation. Further investigations using calcium probes and TMRM staining confirmed that knocking down AHSA1 increased intracellular calcium levels and impaired mitochondrial function in EGFR mutant LUAD cells under Osimertinib stress (Fig. 2E, F and Supplementary Fig. 1J, K). In contrast, overexpression of AHSA1 helped maintain mitochondrial integrity in these cells under the same conditions. Cell cycle assays indicated that silencing AHSA1 forced EGFR mutant LUAD cells into a G1 phase arrest, highlighting AHSA1’s significant role in positively regulating the cell cycle in EGFR mutant LUAD cells (Fig. 2G, H and Supplementary Fig. 2A, B). Overexpression of AHSA1 or enhanced the invasive and migratory capabilities of EGFR mutant LUAD cells, while knocking down AHSA1 impaired these capabilities (Fig. 3A, B and Supplementary Fig. 2C, D). Overexpression of AHSA1 in EGFR-mutant LUAD cells resulted in increased expression of β-catenin and Snail, along with decreased expression of E-cadherin and Vimentin, suggesting that AHSA1 facilitates EMT in EGFR-mutant LUAD cells (Fig. 3E, F). Meanwhile, overexpression of TGFB1 enhances the invasion and migration capabilities of EGFR-mutant LUAD cells. In HEK-293T cells, TGFB1 overexpression increased β-catenin and Snail expression, accompanied by a corresponding downregulation of E-cadherin and Vimentin, indicating that TGFB1 strongly promotes EMT in EGFR-mutant lung adenocarcinoma cells (Fig. 3C, D, G). Tail vein metastasis models demonstrated that AHSA1 overexpression enhanced the metastatic potential of H1650 cells, with simultaneous overexpression of AHSA1 and HSP90AA1 resulting in an even greater number of metastatic lesions. Furthermore, TGFB1 knockdown in this context significantly reduced the metastatic capacity of H1650 cells(Fig. 3H and Supplementary Fig. 3G, H). We thus hypothesize that the AHSA1-HSP90AA1 complex regulates EMT in EGFR-mutant LUAD cells in a TGFB1-dependent manner.

### AHSA1 elevates Akt phosphorylation levels and inhibits CASP3 activation

**Fig. 4:**
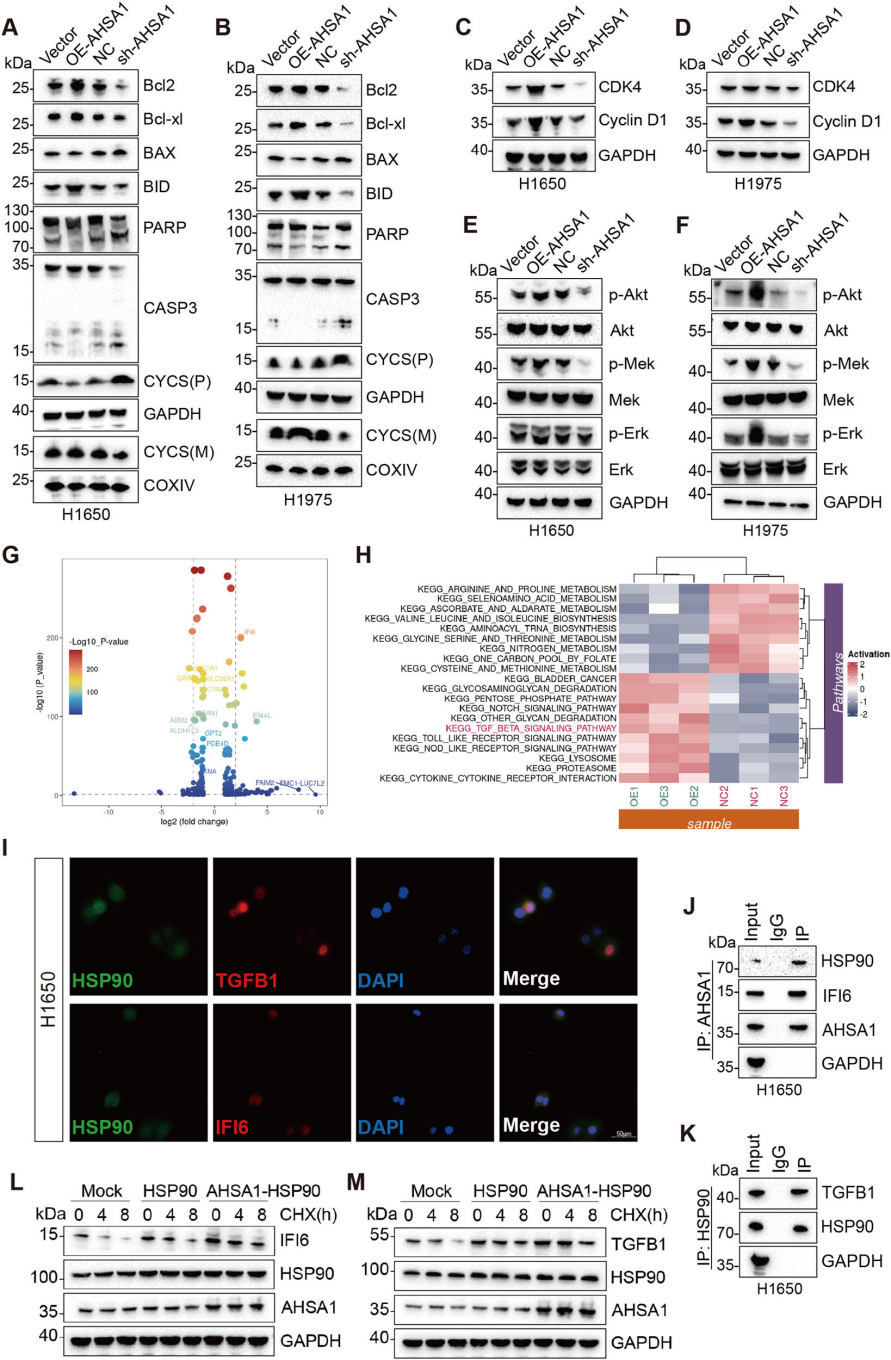
AHSA1 interacts with HSP90AA1 and either IFI6 or TGFB1. **A**, **B** WB was used to measure the expression of apoptosis-related proteins, namely Bcl2, Bcl-xl, BAX, BID, PARP (including cleaved-PARP), CASP3 (including cleaved-CASP3), and CYCS (cytochrome c, both mitochondrial and cytosolic forms). **C**, **D** WB was conducted to assess the expression of cell cycle proteins CDK4 and Cyclin D1. **E**, **F** WB was conducted to assess total and phosphorylated forms of Akt, Mek, and Erk proteins. **G** Based on the transcriptome sequencing performed 48 h after Osimertinib treatment, differentially expressed genes were identified between the AHSA1 overexpressing H1650 cell group and the control group. **H** Gene Set Variation Analysis (GSVA) revealed pathways upregulated in the AHSA1-expressing group compared to the control group. **I** Immunofluorescence was utilized to visualize the cellular localization of HSP90AA1, TGFB1, and IFI. **J**, **K** Co-immunoprecipitation (CO-IP) confirmed the binding interactions among HSP90AA1, AHSA1, and either IFI6 or TGFB1. **L**, **M** Indicated Plasmids were transfected into HEK293T cells, and cell lysates were analyzed by western blotting(WB) at indicated time point. Note: HSP90AA1 abbreviated as HSP90.

WB analysis of EGFR mutant LUAD cells treated with Osimertinib revealed that overexpression of AHSA1 elevated the expression of Bcl2, Bcl-xl, and BID while inhibiting the expression of BAX and the release of mitochondrial CYCS (Fig. 4A, B). Additionally, overexpressing AHSA1 reduced the expression of cleaved forms of PARP and CASP3 in these cells. Concurrently, overexpression of AHSA1 led to increased levels of CDK4, Cyclin D1, beta-catenin, and Snail proteins, and suppressed the expression of E-cadherin (Fig. 4C–F). This overexpression also increased phosphorylation levels of Akt, Mek, and Erk. These findings collectively suggest that AHSA1 can activate the AKT-ERK signaling pathway in EGFR mutant LUAD cells, inhibit apoptosis, promote cell cycle progression, and facilitate epithelial-mesenchymal transition (EMT).

### AHSA1-HSP90AA1 complex interacts with IFI6 and TGFB1

We conducted transcriptome sequencing on H1650 cells overexpressing AHSA1 and control cells both treated with Osimertinib, and GSVA analysis indicated a significant enrichment of the TGF-beta pathway in the AHSA1 overexpression group (Fig. 4G, H). We selected the significantly differentially expressed molecules IFI6 and TGFB1 as potential regulatory targets of AHSA1 for further exploration. Immunoprecipitation and co-immunoprecipitation (CO-IP) results demonstrated that both AHSA1 and HSP90AA1 could bind with IFI6 or TGFB1(Fig. 4I–K and Supplementary Fig. 2G–I). Given the reported role of HSP90AA1 in stabilizing other proteins under cellular stress, we hypothesize that under Osimertinib-induced stress, AHSA1 may enhance the stabilizing effect of HSP90AA1 on IFI6 and TGFB1.

### The AHSA1-HSP90AA1 complex stabilizes IFI6 to regulate Akt phosphorylation levels and Bcl2 expression

**Fig. 5:**
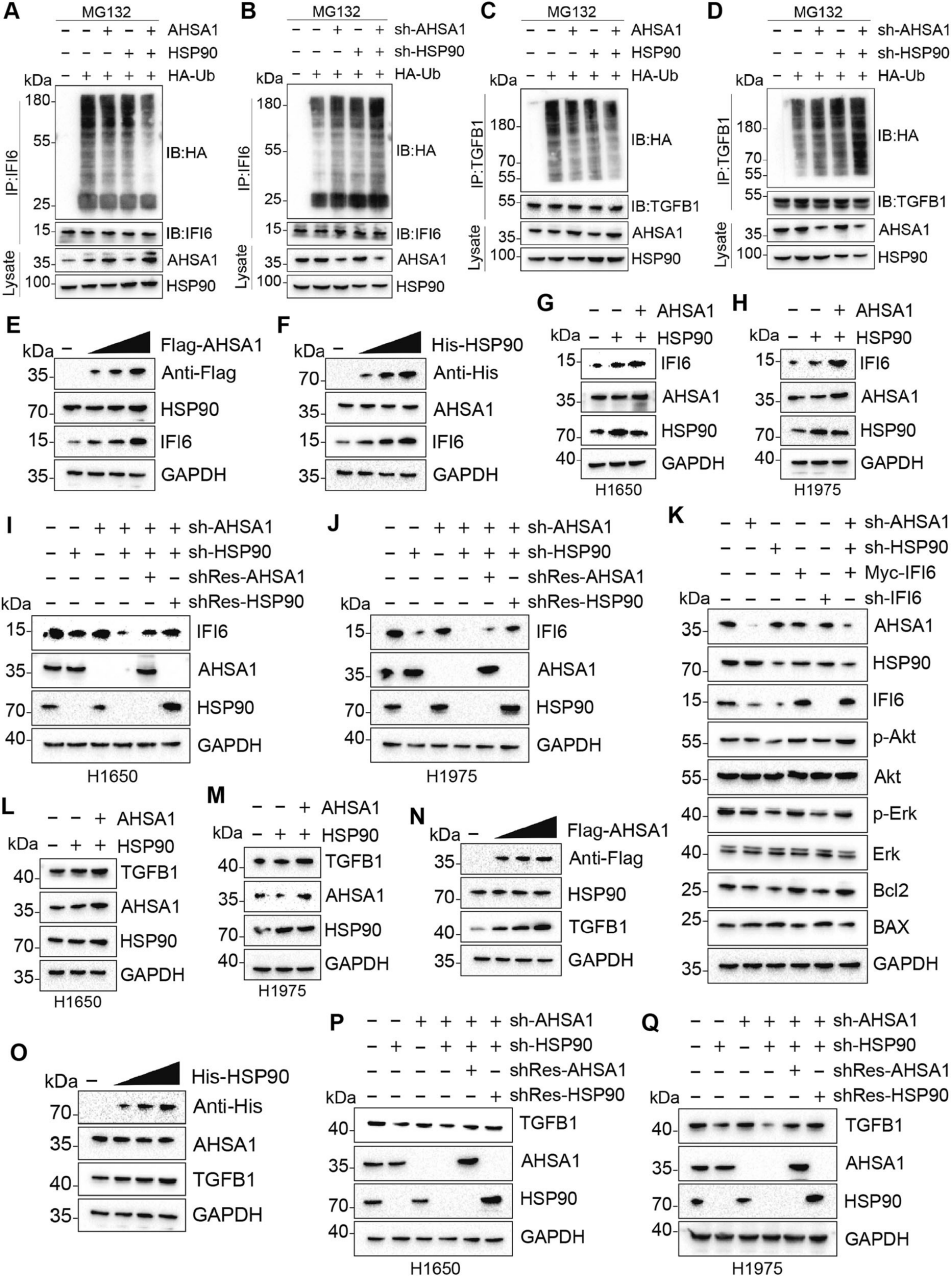
AHSA1 enhanced HSP90AA1 stabilizes IFI6 and TGFB1. **A**–**D** HEK293T cells were co-transfected with Indicated plasmids or sh-RNAs. Cells were treated with MG132 (20 µM, 8 h) before collection. IP of cell lysates was performed using anti-IFI6 antibodies, followed by detection of IFI6 ubiquitination using anti-HA antibodies. WB revealed the effects of manipulating AHSA1 and HSP90AA1 on the expression of IFI6. **E**, **F** WB examined how gradient transfection of AHSA1 or HSP90AA1 impacts IFI6 expression in HEK293T cells. **G**, **H** WB examined how transfection of HSP90AA1 impacts IFI6 expression with or without AHSA1. **I**, **J** The effect of knocking down either AHSA1 or HSP90AA1, as well as simultaneous knockdown of both with subsequent rescue of one, was studied to understand their collective and individual influence on IFI6 levels. **K** The regulation of AHSA1, HSP90AA1, and IFI6 was also analyzed for its impact on the total protein and phosphorylation levels of Akt and Erk, and on the expression of the Bcl2 and BAX proteins. **L**, **M** WB examined how transfection of HSP90AA1 impacts IFI6 expression with or without AHSA1. **N**, **O** WB examined how gradient transfection of AHSA1 or HSP90AA1 impacts TGFB1 expression in HEK293T cells. **P**, **Q** The effect of knocking down either AHSA1 or HSP90AA1, as well as simultaneous knockdown of both with subsequent rescue of one, was studied to understand their collective and individual influence on TGFB1 levels. Note: HSP90AA1 abbreviated as HSP90.

Treating cells with cycloheximide showed that either overexpressing HSP90AA1 alone or both AHSA1 and HSP90AA1, extended the half-life of IFI6 and TGFB1(Fig. 4L, M). Overexpressing HSP90AA1 or AHSA1 alone, or both reduced ubiquitination levels of IFI6 and TGFB1(Fig. 5A, C). Knocking down HSP90AA1 or AHSA1 alone, or both elevated ubiquitination levels of IFI6 and TGFB1(Fig. 5B, D). These findings indicate AHSA1 and HSP90AA1’s role in stabilizing IFI6 and TGFB1.

**Fig. 6:**
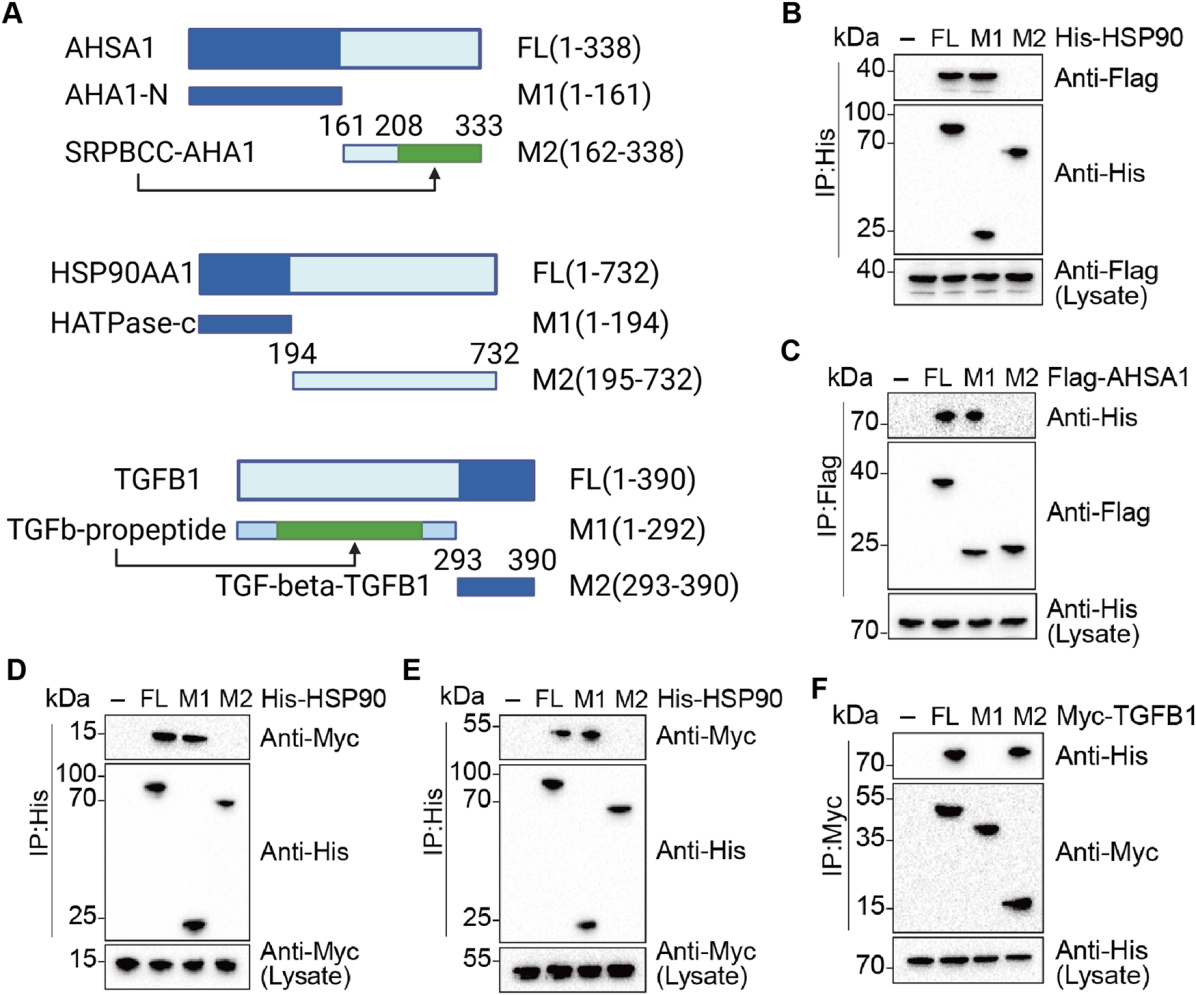
Binding domains where HSP90AA1, AHSA1, IFI6, or TGFB1 interact. **A** Using NCBI’s conserved domain search tool, the structural domains of AHSA1, HSP90AA1, and TGFB1 were identified. Truncation mutants of these proteins were constructed based on these findings. **B** CO-IP of Flag-tagged AHSA1 with His-tagged HSP90AA1 or Its Truncation Mutants. **C** Co-IP of His-tagged HSP90AA1 with Flag-tagged AHSA1 or Its Truncation Mutants. **D** Co-Immunoprecipitation of Myc-tagged IFI6 with His-tagged HSP90AA1 or Its Truncation Mutants. **E** Co-Immunoprecipitation of Myc-tagged TGFB1 with His-tagged HSP90AA1 or Its Truncation Mutants. **F** Co-Immunoprecipitation of His-tagged HSP90AA1 with Myc-tagged TGFB1 or Its Truncation Mutants. Note: HSP90AA1 abbreviated as HSP90.

AHSA1 and HSP90AA1’s modulation of IFI6 expression were dose-dependent (Fig. 5E, F). Overexpression of HSP90AA1 increased IFI6 protein expression, and this increase was further enhanced by the co-overexpression of AHSA1 and HSP90AA1(Fig. 5G, H). Knocking down either HSP90AA1 or AHSA1 reduced IFI6 protein expression, and the decrease in IFI6 expression caused by knocking down both AHSA1 and HSP90AA1 could be rescued by shRNA against HSP90AA1, but not by shRNA against AHSA1(Fig. 5I, J). These findings suggest that AHSA1 collaboratively enhances IFI6 protein expression with HSP90AA1, and the promotive effect of AHSA1 on IFI6 expression is dependent on HSP90AA1. Further experiments demonstrated that overexpression of IFI6 could mitigate the effects of knocking down HSP90AA1 and AHSA1, elevating the phosphorylation levels of Akt and Erk, increasing Bcl2 expression, and decreasing Bax expression (Fig. 5K). After using conserved domain prediction, truncated mutants of HSP90AA1, AHSA1, and IFI6 were constructed (Fig. 6A). Immunoprecipitation results indicated that the M1 domain of AHSA1 could bind to HSP90AA1, and the M1 domain of HSP90AA1 could bind to either AHSA1 or IFI6(Fig. 6B–D). Thus, it is hypothesized that the M1 domains of AHSA1, the M1 domains of HSP90AA1, and IFI6 interact to exert their effects.

### AHSA1-HSP90AA1 complex maintains TGFB1 stability

Overexpression of HSP90AA1 led to an increase in TGFB1 protein expression, which was further enhanced by the co-overexpression of AHSA1 and HSP90AA1 (Fig. 5L, M). AHSA1 and HSP90AA1’s modulation of IFI6 expression were dose-dependent (Fig. 5N, O).

Knocking down either HSP90AA1 or AHSA1 reduced TGFB1 protein expression, and the reduction in TGFB1 expression caused by knocking down both AHSA1 and HSP90AA1 could be rescued by shRNA against HSP90AA1 but not by shRNA against AHSA1 (Fig. 5P, Q). These results suggest that AHSA1 collaborates with HSP90AA1 to promote TGFB1 protein expression, and the enhancement of TGFB1 expression by AHSA1 is dependent on HSP90AA1. The regulation of TGFB1 after manipulating AHSA1 and HSP90AA1 was similar to the changes observed with IFI6, suggesting that AHSA1 and HSP90AA1 may similarly regulate the stability of TGFB1. After using conserved domain prediction, a truncated mutant of TGFB1 was constructed and subjected to immunoprecipitation with the previously mentioned truncated mutant of HSP90AA1 (Fig. 6A). The results showed that the M1 domain of HSP90AA1 could bind to the M2 domain of TGFB1 (Fig. 6E, F). Based on these findings, it is hypothesized that the M1 domain of AHSA1, the M1 domain of HSP90AA1, and the M2 domain of TGFB1 interact to exert their biological effects

### IFI6 regulates cell survival under Osimertinib stress as the crucial downstream effector of the AHSA1-HSP90AA1 complex

**Fig. 7:**
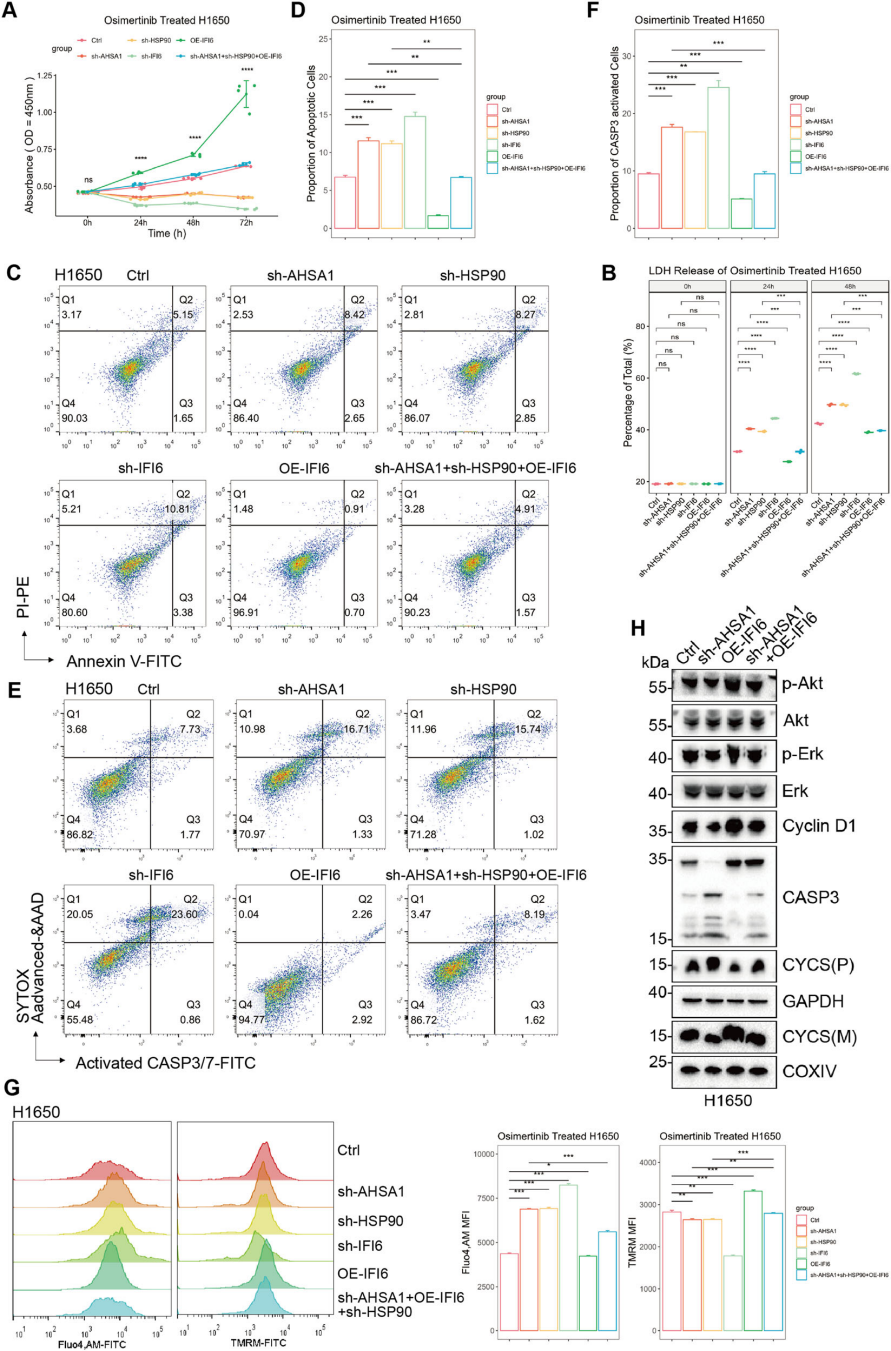
HSP90AA1-AHSA1 complex relies on IFI6 to control apoptosis evasion and mitochondrial stability. **A**, **B** Cell viability in stably transfected cell lines (sh-AHSA1, sh-HSP90AA1, sh-IFI6, OE-IFI6, and sh-AHSA1+sh-HSP90AA1 + OE-IFI6 and Ctrl) under Osimertinib stress was measured using the CCK-8 assay, and apoptosis was assessed with the LDH assay. **C**–**F** Under Osimertinib stress, the apoptosis and CASP3/7 activation status in stably transfected cell lines were assessed. **G** The concentration of intracellular free calcium ions in these cell lines under Osimertinib stress was measured using Fluo-4, AM probe, and Mitochondrial activity was examined using TMRM staining. **H** WB was used to measure the expression of total and phosphorylated forms of Akt, and Erk proteins, as well as CDK4, CASP3 (including cleaved-CASP3), and CYCS (cytochrome c, both mitochondrial and cytosolic forms) proteins in H1650 cells. Note: *n* = 3, Error bars represent the mean ± SD. *P < 0.05, **P < 0.01, ***P < 0.001, ****P < 0.0001.

Upon manipulating AHSA1, HSP90AA1, and IFI6, we assessed the apoptosis and mitochondrial function in EGFR mutant LUAD cells under the stress of Osimertinib. Knocking down AHSA1, HSP90AA1, and IFI6 significantly increased apoptosis and elevated the expression of active CASP3/7 in these cells (Fig. 7A–F and Supplementary Fig. 3A–F). Conversely, overexpressing IFI6 conferred resistance to apoptosis in EGFR mutant LUAD cells under Osimertinib stress and could partially rescue the effects of simultaneously knocking down AHSA1 and HSP90AA1. Knocking down AHSA1, HSP90AA1, and IFI6 significantly impaired mitochondrial stability in these cells (Fig. 7G and Supplementary Fig. 4A, B). Conversely, overexpressing IFI6 maintained mitochondrial stability in EGFR mutant LUAD cells under Osimertinib stress and could partially rescue the effects of simultaneously knocking down AHSA1 and HSP90AA1. WB analysis validated these findings at the protein level (Fig. 7H and Supplementary Fig. 4C). Thus, IFI6 emerges as a crucial downstream molecule necessary for the anti-stress effects of AHSA1 and HSP90AA1, maintaining cell survival under the pressure of Osimertinib.

### IFI6 promotes cell growth and Osimertinib resistance in vivo

**Fig. 8:**
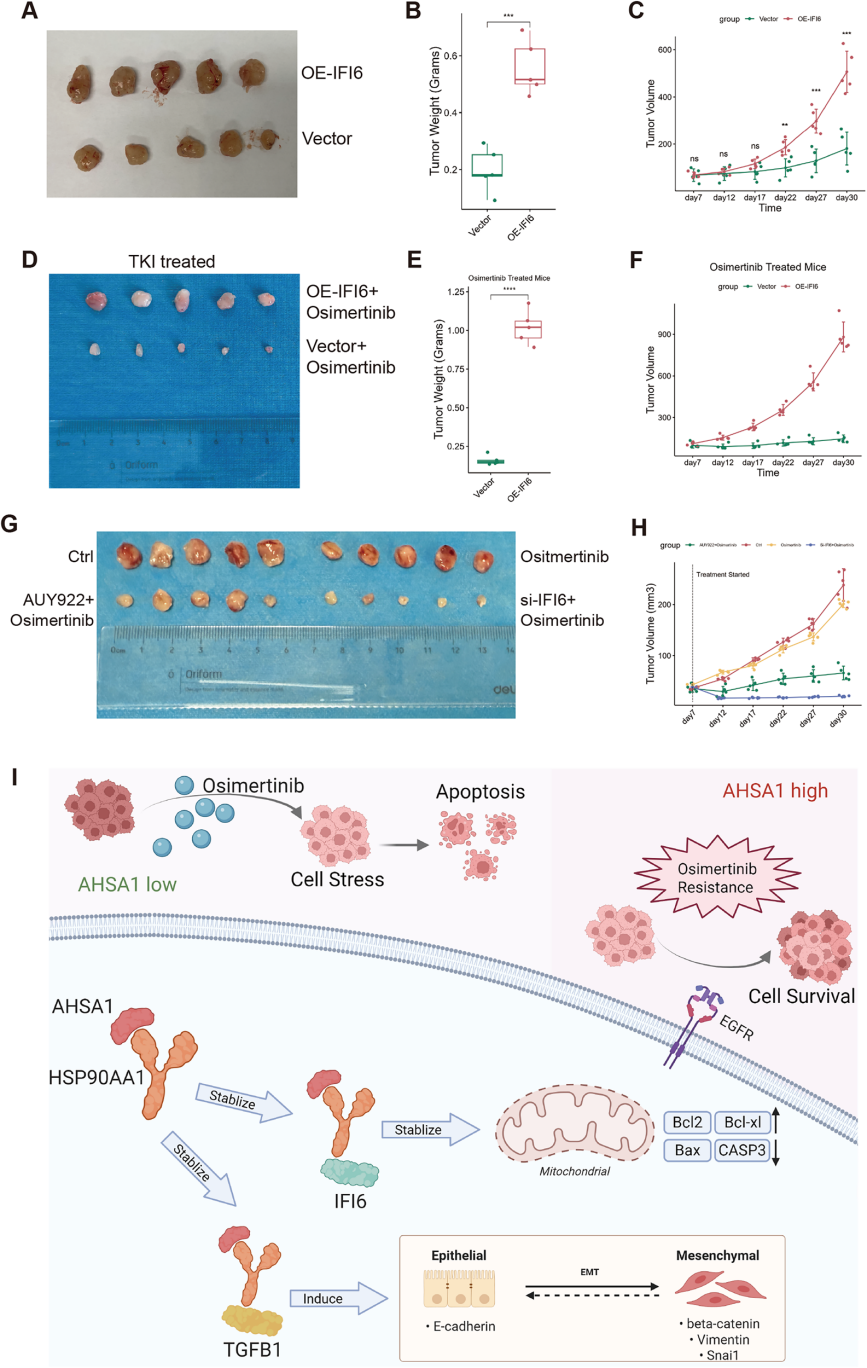
IFI6 promotes tumor growth and Osimertinib resistance in vivo. **A–F** Under normal conditions and with Osimertinib treatment, the subcutaneous tumor formation in nude mice with H1650 cells overexpressing IFI6 and in the control group is observed. **G**, **H** The subcutaneous tumor model based on HCC827-TS was used to evaluate the efficacy of Osimertinib, AUY922 + Osimertinib, and Si-IFI6 + Osimertinib. **I** Illustration of the mechanism studied in this research. Note: HSP90AA1 abbreviated as HSP90. *n* = 5, Error bars represent the mean ± SD. *P < 0.05, **P < 0.01, ***P < 0.001, ****P < 0.0001.

The subcutaneous xenograft experiment further substantiated our findings. Under normal conditions, the group overexpressing IFI6 exhibited increased growth and weight of subcutaneous tumors derived from H1650 stable transfected cells compared to the control group (Fig. 8A–C and Supplementary Fig. 4D). One week after bilateral implantation of the IFI6-overexpressing group and control H1650 cells into the same nude mice, oral Osimertinib treatment was administered 5 days a week for 3 weeks. The tumors in the control group visibly regressed, whereas those in the IFI6-overexpressing group continued to grow (Fig. 8D–F and Supplementary Fig. 4E). Subcutaneous tumor experiments based on the Osimertinib-resistant HCC827-TS cell line revealed that the HSP90 inhibitor AUY922 demonstrated considerable efficacy against Osimertinib-resistant EGFR-mutant LUAD, with IFI6 knockdown showing even greater therapeutic potential in vivo (Fig. 8G, H and Supplementary Fig. 4F). These findings collectively demonstrate that IFI6 promotes Osimertinib resistance in EGFR-mutant LUAD in vivo and represents a potential therapeutic target. The mechanism studied in this research is illustrated in Fig. 8I.

## Discussion

In this study, we have demonstrated for the first time that IFI6 is a principal downstream molecule through which the AHSA1-HSP90AA1 complex promotes resistance to Osimertinib and stabilizes mitochondria in EGFR mutant LUAD. We also showed that the AHSA1-HSP90AA1 complex may promote EMT in EGFR mutant LUAD by stabilizing TGFB1. While most existing research has focused on HSP90AA1 and its activators like AHSA1 or inhibitors like CDC37, revealing important mechanisms by which HSP90AA1 and interacting proteins such as HSP70 maintain cellular homeostasis and proliferative activity under stress [20–23], the resultant HSP90AA1 inhibitor drugs have offered new hope for the treatment of various cancers, including lung cancer, especially after the onset of drug resistance [24, 25]. However, the side effects and toxicity caused by inhibiting HSP90AA1 and a broad spectrum of downstream molecules cannot be overlooked. As early as 2015, Melissa L. Johnson et al. reported a Phase I/II study on the treatment of EGFR-mutant lung cancer patients using the HSP90 inhibitor AUY922 in combination with erlotinib. While partial responses were observed in some patients, the therapy failed to achieve its predefined primary endpoint due to toxicity, particularly night blindness [26]. Subsequently, AUY922 was again reported to be associated with severe adverse effects such as diarrhea, nausea, and decreased appetite in the treatment of EGFR-mutant NSCLC patients [27]. Similarly, the NCT01646125 study investigated the efficacy of AUY922 compared to pemetrexed or docetaxel in NSCLC patients with EGFR mutations. The AUY922 arm demonstrated significantly higher rates of severe and overall adverse effects compared to the chemotherapy arm. These studies suggested that specifically targeting downstream effectors of HSP90AA1 might be a superior strategy.

IFI6, an apoptosis-regulating molecule, is significantly upregulated under AHSA1 overexpression and stabilizes the mitochondria in EGFR mutant LUAD cells under Osimertinib stress, while also inhibiting CASP3 activation, positioning it as a major downstream effector of HSP90AA1 in regulating cellular homeostasis under stress. Under the stabilization by the AHSA1-HSP90AA1 complex, the expression of IFI6 not only robustly activates the expression of the BCL2 family to inhibit apoptosis but also enhances the phosphorylation levels of AKT, thereby promoting the survival of EGFR mutant LUAD cells under Osimertinib stress [28, 29]. This offers a novel mechanistic explanation for resistance to Osimertinib [30].

Furthermore, our research indicates that upregulation of AHSA1 activates the TGF-beta pathway in EGFR mutant LUAD under Osimertinib stress. Previous studies have shown that TGFB1 signaling controls SMAD2/3 and directs their binding to targets to exert functions [31, 32]. Research has reported TGFB1-mediated activation of Snail or the regulation of other molecules like Blimp1, which promotes EMT [33, 34]. Our findings reveal that upregulation of AHSA1 enhances the invasive and migratory capabilities of EGFR mutant LUAD under Osimertinib stress and leads to the loss of epithelial characteristics, suggesting that TGFB1 stabilized by the AHSA1-HSP90AA1 complex might be a primary driver of EMT in EGFR mutant LUAD.

The occurrence of EMT under TKI stress not only undermines the efficacy of TKI therapy but also engenders resistance to subsequent PD-1/PD-L1 treatments [35–37], presenting a significant barrier in the treatment of EGFR mutant LUAD [38]. Existing research has identified that LUAD cells exhibiting high CD70 expression post-EMT form a persistent drug-resistant subpopulation, leading to TKI treatment failure [39]. Considering that EMT contributes to TKI resistance, TGFB1, when stabilized by HSP90AA1 in this study, may also promote EMT in EGFR mutant LUAD, thus intensifying resistance to Osimertinib. However, given that the overexpression of IFI6 robustly reversed the detrimental effects on survival caused by knocking down AHSA1 and HSP90AA1 in EGFR mutant LUAD cells, our study suggests that IFI6 is the principal player in mediating AHSA1-HSP90AA1 regulation and promoting resistance to Osimertinib.

Our study uncovered a critical mechanism of Osimertinib resistance in the subset of patients with high AHSA1 expression. However, the substantial heterogeneity among patients presents a significant challenge. The varying expression of AHSA1 across different lesions within the same patient and its impact on the translational relevance of this study remain uncertain. This limitation highlights the need for subsequent validation through extensive population-based cohorts to confirm our findings. Future research on the sustained active expression or inhibition of IFI6 and similar downstream molecules associated with HSP90AA1 in EGFR mutant LUAD and other cancer subtypes is also crucial for advancing our understanding of tumor stress responses and resistance.

## Methods and materials

Protein stability assays, IHC, tail vein metastasis models and in vivo ubiquitination experiments have been previously detailed [40].

### Cell lines and cell culture

The LUAD cell lines (H1650, H1975, HCC827) were provided by the Cell Repository of the Chinese Academy of Sciences located in Shanghai. The cells were cultivated in Roswell Park Memorial Institute medium (RPMI) 1640 (Gibco, USA), which was supplemented with 10% fetal bovine serum (FBS) (FBSKM0503, Ozfan, China). The Osimertinib-resistant cell line HCC827-TS, derived from HCC827, was kindly provided by Prof. Yin from the Yin Lab (Jiangsu Cancer Hospital, China).

### Patient specimen

Human LUAD tissues were collected from the sample library of the Affiliated Cancer Hospital of Nanjing Medical University. The samples were frozen and stored in liquid nitrogen. The diagnosis of LUAD was confirmed based on clinical manifestation and pathological examination. Informed consent was obtained from all subjects.

### CCK-8 Assay

At the designated time, selected wells were augmented with CCK-8 reagent (APExBIO, USA). Following a 1-h incubation in the culture incubator, a microplate reader (ELx808, Bio Tek, USA) was utilized to assess absorbance values at 450 nm.

### Lactate dehydrogenase(LDH) Assay

The supernatant group received 20 μL of distilled water before wells were gently pipetted and mixed, while the total LDH group received 20 μL of lysis buffer. The plate was incubated for 45 min. Following that, the supernatant was put in a new 96-well tray with an equal quantity of the reaction mixture (Invitrogen, USA). A stop solution was added 30 min later, and absorbance values at 490 nm and 630 nm were recorded. The absorbance at 630 nm was used as the background value.

### EdU assay

The EdU working solution (Beyotime, China) was preheated and added to the six-well plate. Afterwards, each cell was fixed with 4% paraformaldehyde (15 min, 25 °C). Afterward, each well was washed with Phosphate Buffered Saline (PBS) (KeyGEN BioTECH, China) and permeabilized with PBS having 0.3% Triton X-100 (Beyotime, China). Following permeabilization, each well was washed with PBS and covered with the reaction solution. The plate was stirred lightly and placed at 25 °C to 30 min in darkness. Finally, the reaction solution was aspirated, and washing of cells was performed two times using cell stain buffer(Biolegend, USA) before analysis by BD FACSCelesta flow cytometer (BD Bioscience, USA). FlowJo V10 was employed to determine the results.

### Annexin V and propidium iodide(PI) staining

Cells were washed and reconstituted in a Binding Buffer. Following that, each tube received a mix of FITC-Annexin V antibodies and Propidium Iodide (PI) Solutions (APExBIO, Houston, USA) respectively, with subsequent dark incubation spanning for 10 min. Then, each tube was filled with Binding Buffer, then samples were analyzed through a flow cytometer and FlowJo V10 was used to assess the findings.

### Activated caspase-3/7 and SYTOX AADvanced staining

Cells were washed and resuspended in cell stain buffer. Subsequently, each tube was added with Caspase-3/7 Green Reagent(Invitrogen, USA), and incubation was conducted at 37 °C in a light-protected atmosphere (with 5% CO_2_, 25 min). Afterward, SYTOX AADvanced(Invitrogen, USA) was added, and incubation lasted 5 min. Finally, the specimens were run using a flow cytometer, and FlowJo V10 software was employed for data analysis.

### Mitochondrial membrane potential integrity assay

Cells were extracted and treated with 1 mL of cell stain buffer, and Image-iT TMRM Reagent (Invitrogen, USA) was added to each tube. Thereafter, incubation was conducted at 37 °C in a light-protected environment for 30 min. FlowJo V10 was used for data interpretation after the sample analysis through a flow cytometer.

### Calcium ion fluorescent probe assay

Cells were washed with HBSS (Hank’s Balanced Salt Solution) (KeyGEN BioTECH, China) solution. Fluo-4 AM (Yeasen, China) working fluid is added to these cells for a final concentration of 1 μM. Then, incubation was conducted for 30 min at 37 °C. After washing and resuspension in the HBSS solution, incubation was performed for another 30 min. FlowJo V10 was used for data interpretation after the sample analysis through the flow cytometer.

### Cell cycle analysis

Cells were washed and submerged in 75% ethanol overnight at −20 °C. Fixative extracted, a ready-to-use PI staining solution containing RNase (Invitrogen, USA) was used. After incubating the samples (37 °C, 30 min), the labeled cells passed through using a flow cytometer, and the data was processed with Modfit 5.

### Construction of stable AHSA1 knock-down and AHSA1 over-expressing EGFR-mutated lung adenocarcinoma cell lines

The AHSA1 over-expression or knock-down lentivirus, purchased from Obio Tech (Shanghai, China), was used to transfect H1650 and H1975 cells. Subsequently, the cells remained continuously refined in a medium holding 2 μg/mL puromycin (Gibco, USA) to obtain stably transfected cell lines. The efficiency of AHSA1 knock-down or over-expression was studied using WB analysis.

### Tumor xenografts

The cells were rinsed 3 times with PBS. They were resuspended in a mixture of PBS and Matrigel (Corning, USA). The cells were then injected under the skin of 5-week-old BALB/c nude mice(GemPharmath, China). For normal experiment, each mouse was inoculated with 5 × 10^6^ cells in a 100 µL volume. For experiment with oral Osimertinib, each mouse was inoculated with 1 × 10^7^ cells in a 100 µL volume. The extent of the tumor was calculated on a 5-day basis.

Mice treated with Osimertinib (Targetmol, Shanghai, China) followed a regimen of oral gavage five times per week for four weeks (5 mg/kg). Mice treated with AUY922 received intraperitoneal injections every other day for 4 weeks (50 mg/kg). Mice treated with in vivo Si-IFI6 received localized injections following the manufacturer’s recommended dosage, administered three times per week for 4 weeks.

All of the mice were mercifully murdered with CO_2_ at the designated time point, and the subcutaneous tumors were removed for size measurement. The cancer’s volume was approximated by a formula such as: (length × width^2^)/2.

### Co-immunoprecipitation assay

Cells were washed and then immersed in pre-chilled IP lysis buffer (Thermo Scientific, USA) containing a protease inhibitor cocktail (Sigma, USA). After lysis on ice for 30 min, the lysate was subsequently centrifuged at 4 °C, 12,000 × *g* for 15 min, and the supernatant was transferred to a new centrifuge tube for measurement of protein concentration. Magnetic beads (Millipore, USA) were collected and rinsed repeatedly using an IP lysis solution before being incubated with selected antibodies. Afterward, an appropriate volume of protein supernatant was added, and incubation was carried out overnight on a rotating shaker at 4 °C. The magnetic beads were extracted the next day by the magnet holder and cleansed three times with a washing solution, followed by a rinse with pure water. Finally, the proteins were eluted using an Elution Buffer for 3 min, and SDS (Sodium Dodecyl Sulfate) buffer (Beyotime, China) was poured and then heated at 95 °C in a metal bath for 5 min. WB was performed to analyze the protein components.

### Transcriptome sequencing and analysis

Following collection, overexpression of AHSA1 and control H1650 cells were resuspended in Trizol and utilized for sequencing. The transcriptome sequencing was provided by the company lc-bio(China). After quality control and filtering, the Fastq files were quantified using featureCounts software and subjected to subsequent analysis in R software (version = 4.3.2). DESeq2 package (version = 1.42.1) was employed for inter-group differential analysis, while enrichment analysis utilized the clusterProfiler package (version = 4.10.1). The mRNA sequencing raw data has been uploaded into the SRA database, and the BioProject is PRJNA1245493.

### Public data download and analysis

The TCGA-LUAD data was downloaded, cleaned, and analyzed using the R package TCGAbiolinks (version = 2.30.0). Survival analysis was conducted utilizing the R packages survival (version = 3.5-8) and survminer (version = 0.4.9).

### Western blot analysis

After lysing cells with lysis buffer containing protease and phosphatase inhibitors of protein extraction kit (EX1100, Solarbio, China). For 20 min, the blood lysates were kept on ice. They were then spun up at 12,000 × *g* for 15 min at 4 °C, and the remaining residues were taken out to be measured with a spectrophotometer (NanoDrop One, Thermo Scientific, USA). Following that, an SDS sample buffer was inserted, and it was put over a metal bath at 95 °C for 10 min.

These were loaded into a precast gel (GenScript, China) and subjected to constant voltage electrophoresis until the bromophenol blue reached its bottom. After that, proteins were moved into a PVDF sheet (Millipore, USA) using a wet transfer system. Following this, the PVDF membrane was blocked with 5% BSA (Solarbio, China) for 30 min and then cut into appropriate sections for antibody incubation at 4 °C overnight. Afterward, the membrane was washed in 1X PBST (NCM Biotech, China) on a shaker and subsequently incubated with the corresponding secondary antibodies. After another wash with 1X PBST on a shaker, the membrane was developed using an ECL substrate (Millipore, USA) and subjected to detection using a Chemiluminescence imaging system (Universal Hood II, BIO-RAD, USA).

### Statistical analysis and data visualization

The data were presented as the mean ± standard deviation. The experiments were replicated three times. The programming system R was used for data analysis (version = 4.3.2). *T*-tests, one-way ANOVA, and to compare variations among multiple groups, a two-sided ANOVA was applied. When a value of *p* < 0.05 was chosen, the significance level was assessed. The R packages were utilized to present the information, ggplot2 (version = 3.4.3) and ggpubr, and data preprocessing was done using the dplyr (version = 1.1.2) and tidyverse (version = 2.0.0) packages.

## Supplementary information

Supplementary figures

Full and uncropped western blots

Antibody list

## Data Availability

The data that support the findings of this study are available from the corresponding author upon reasonable request.
