# Supplementary material for: AHSA1-HSP90AA1 complex stabilized IFI6 and TGFB1 promotes mitochondrial stability and EMT in EGFR-mutated lung adenocarcinoma under Osimertinib pressure
Source: Cell Death Dis. 2025 Apr 15;16(1):298. doi: 10.1038/s41419-025-07650-9 (PMC12000569; doi:10.1038/s41419-025-07650-9)
Supplement: Supplementary file 1 — Supplementary figures [file 41419_2025_7650_MOESM1_ESM.pdf]

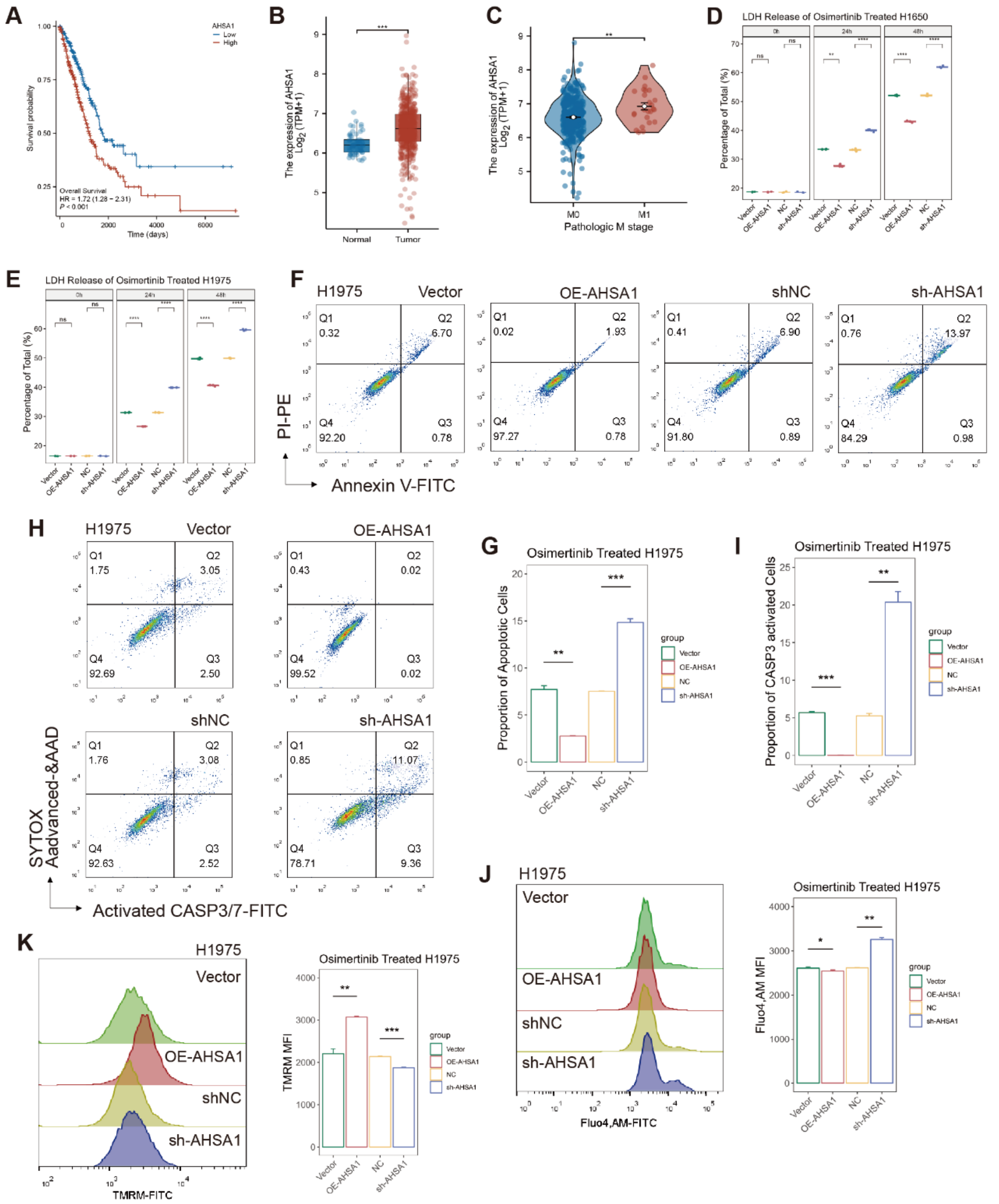

Supplement Fig. 1 A The overall survival time of TGCA-LUAD patients regarding the expression of AHSA1 (top and bottom 50% based on median expression). **B** The expression of AHSA1 in cancerous tissues and adjacent normal tissues in TCGA-LUAD data. **C** The expression of AHSA1 in patients with metastatic and non-metastatic lung adenocarcinoma in the TGCA-LUAD dataset. **D-E** Apoptosis was assessed with the LDH assay **F-I** Under Osimertinib stress, the apoptosis and CASP3/7 activation status in stably transfected cell lines were assessed. **J** The concentration of intracellular free calcium ions in these cell lines under Osimertinib stress was measured using Fluo-4, AM probe. **K** Mitochondrial activity was examined using TMRM staining. **Note:** n=3, Error bars represent the mean ±S.D. *P < 0.05, **P < 0.01, ***P < 0.001, ****P<0.0001.

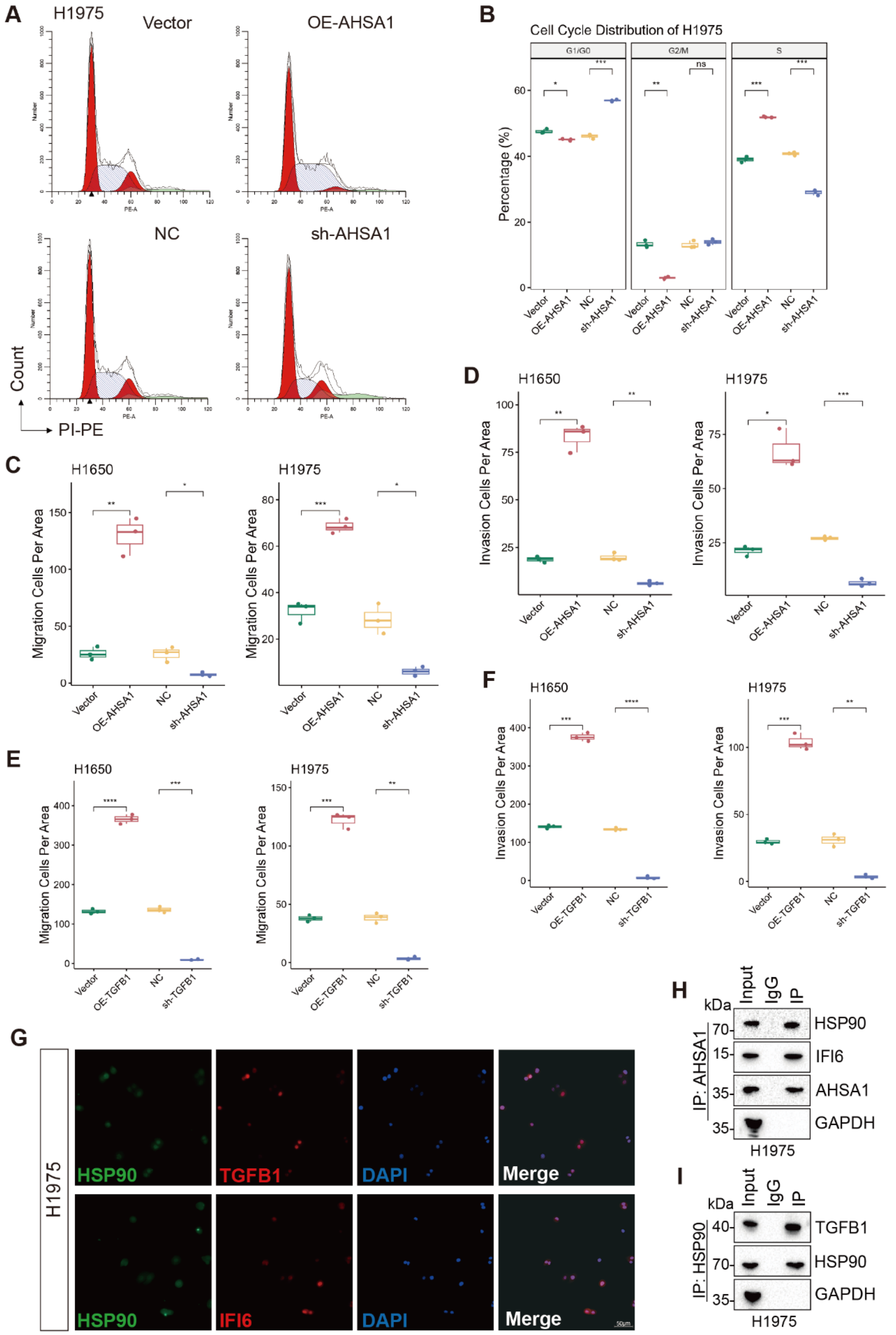

Supplement Fig. 2 A-B The distribution of cell cycle phases in the stably transfected cell lines was analyzed using PI staining. **C-F** Statistical analysis of invasion and migration experiments. **G** immunofluorescence was utilized to visualize the cellular localization of HSP90AA1, TGFB1,

and IFI6. **H-I** CO-IP confirmed the binding interactions among HSP90AA1, AHSA1, and either IFI6 or TGFB1 **Note:** HSP90AA1 abbreviated as HSP90. n=3, Error bars represent the mean ±S.D. *P < 0.05, **P < 0.01, ***P < 0.001, ****P<0.0001.

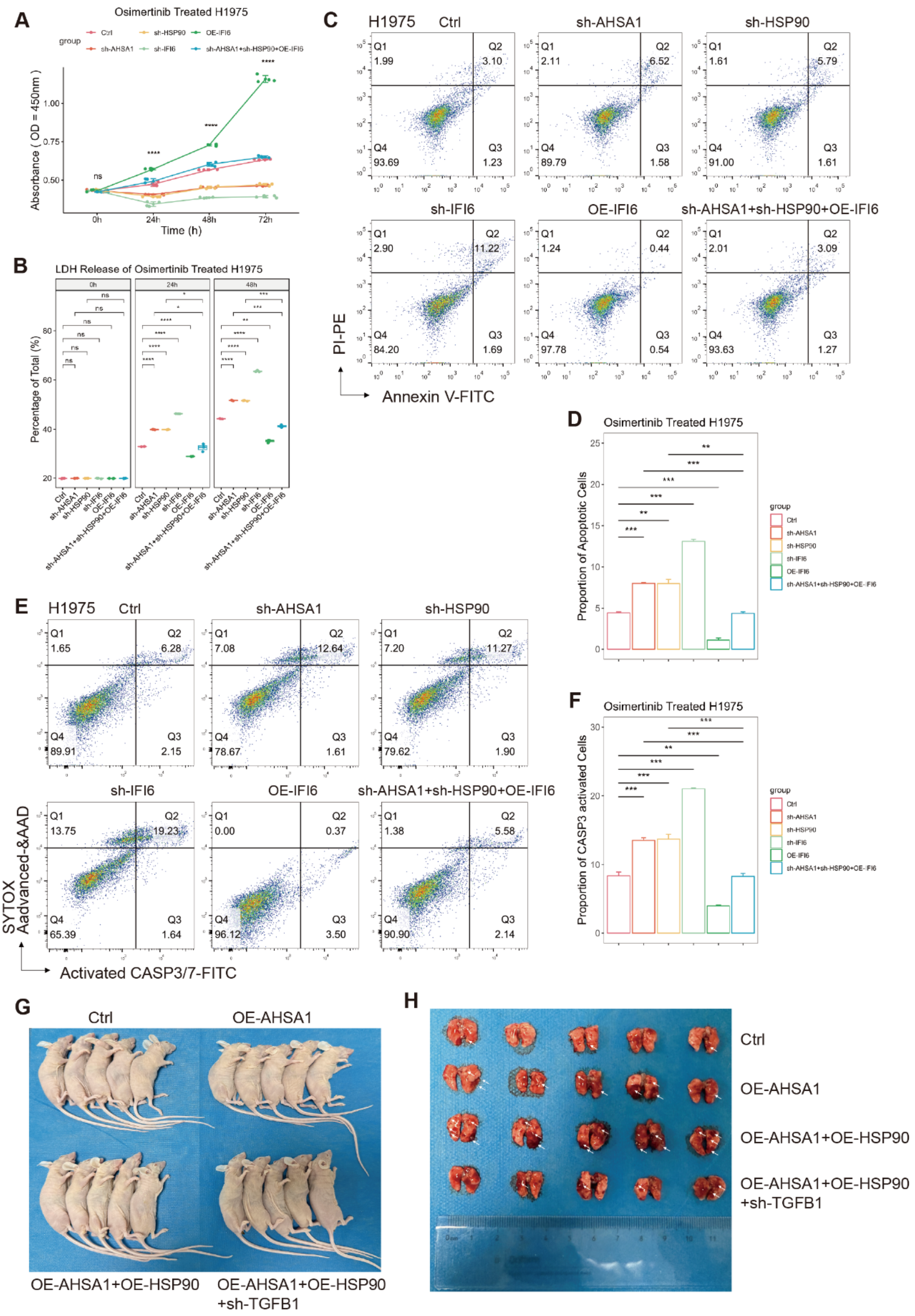

Supplement Fig. 3 A-B Cell viability in stably transfected cell lines(sh-AHSA1, sh-HSP90AA1,

sh-IFI6, OE-IFI6, and sh-AHSA1+sh-HSP90AA1+OE-IFI6 and Ctrl) under Osimertinib stress was measured using the CCK-8 assay, and apoptosis was assessed with the LDH assay. **C-F** Under Osimertinib stress, the apoptosis and CASP3/7 activation status in stably transfected cell lines were assessed. **G-H** Group presentation of mice and lung metastatic lesions in the tail vein lung metastasis models. **Note:** HSP90AA1 abbreviated as HSP90. n=3, Error bars represent the mean ±S.D. *P < 0.05, **P < 0.01, ***P < 0.001, ****P<0.0001.

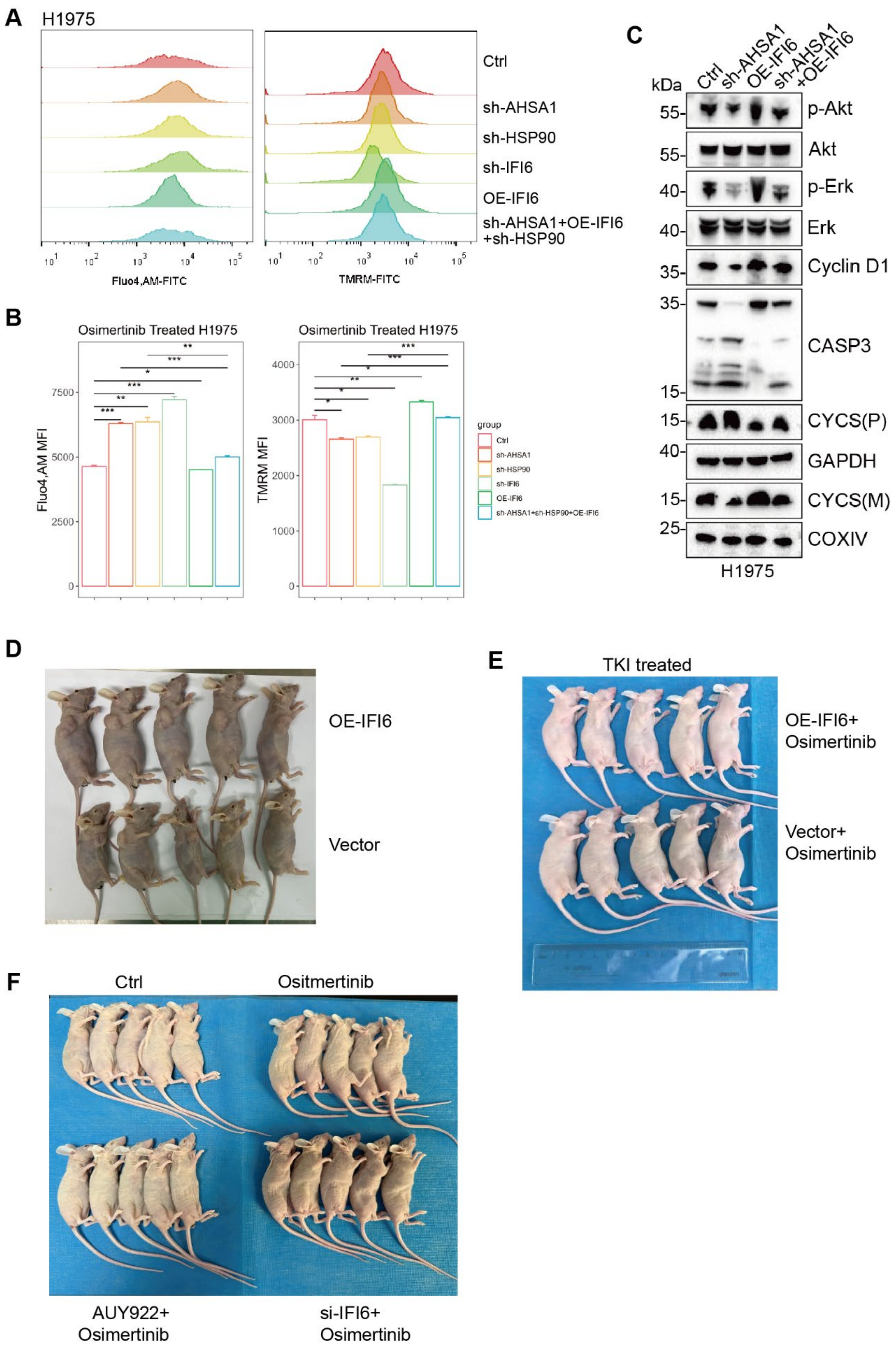

Supplement Fig. 4 A-B The concentration of intracellular free calcium ions in these cell lines under Osimertinib stress was measured using Fluo-4, AM probe, and Mitochondrial activity was examined using TMRM staining. **C** WB was used to measure the expression of total and

phosphorylated forms of Akt, and Erk proteins, as well as CDK4, CASP3 (including cleaved-CASP3), and CYCS (cytochrome c, both mitochondrial and cytosolic forms) proteins in H1975 cells. **D-F** Photos of mice with subcutaneous tumors.
