# Supplementary material for: AHSA1-HSP90AA1 complex stabilized IFI6 and TGFB1 promotes mitochondrial stability and EMT in EGFR-mutated lung adenocarcinoma under Osimertinib pressure
Source: Cell Death Dis. 2025 Apr 15;16(1):298. doi: 10.1038/s41419-025-07650-9 (PMC12000569; doi:10.1038/s41419-025-07650-9)

Figure1D

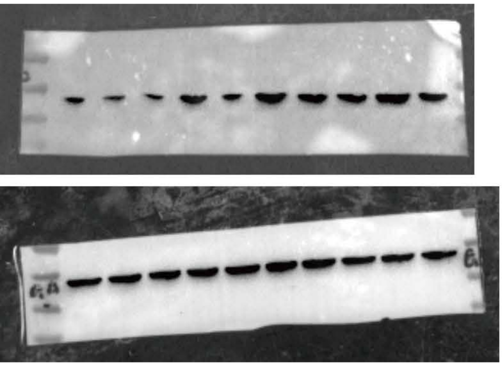

Figure1H

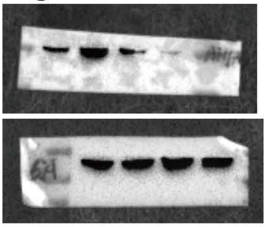

Figure1I

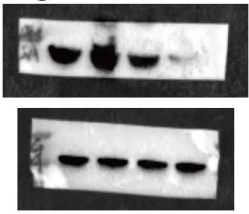

Figure1G

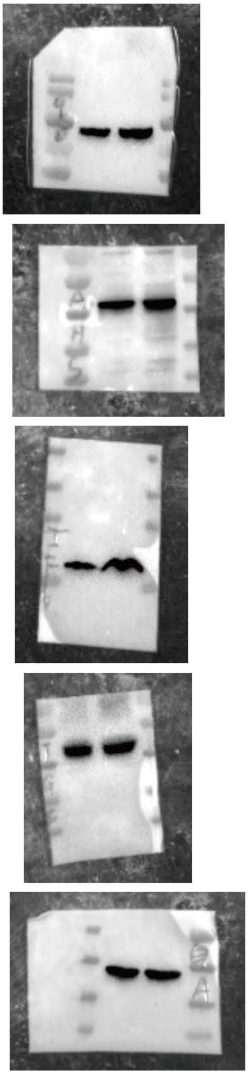

Figure3E

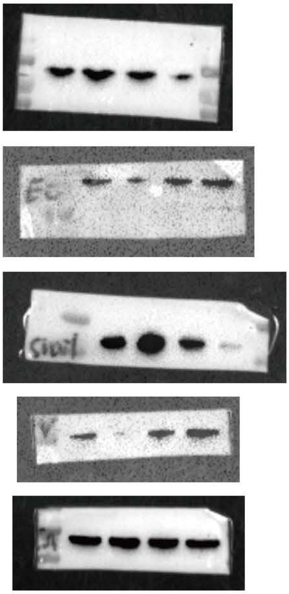

Figure3F

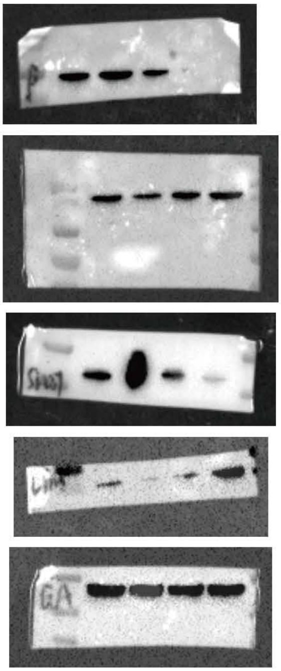

Figure3G

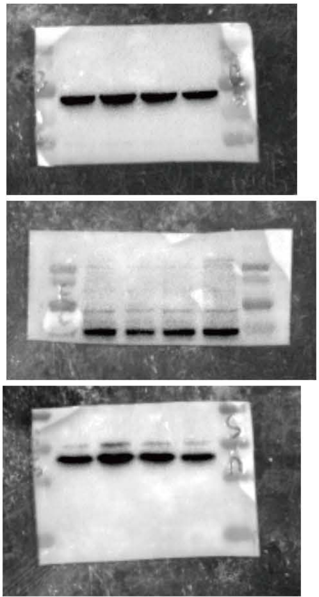

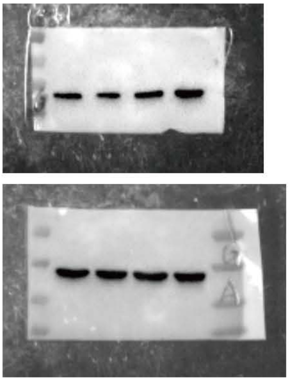

Figure4A

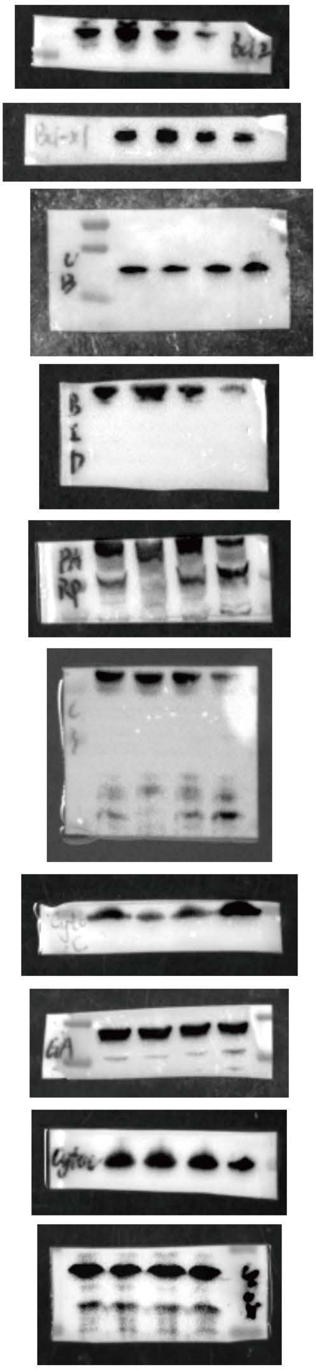

Figure4B

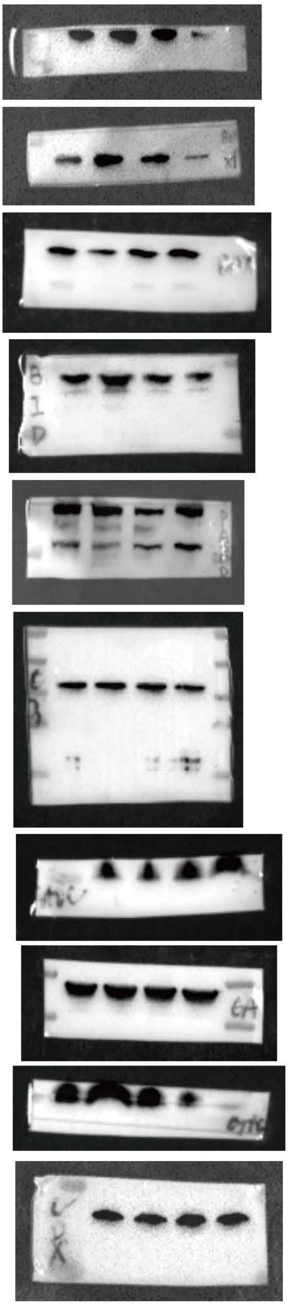

Figure4C

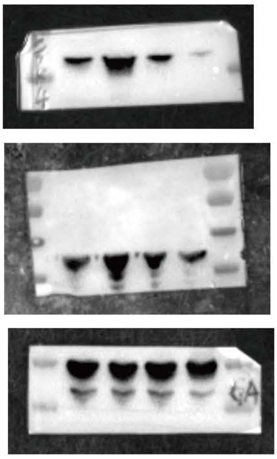

Figure4D

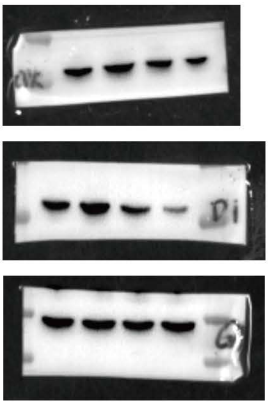

Figure4E

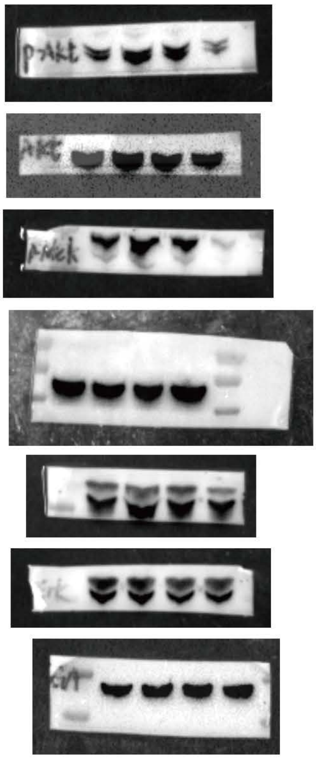

Figure4F

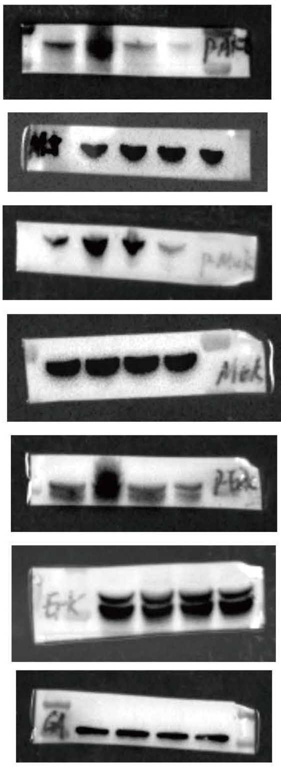

Figure4B

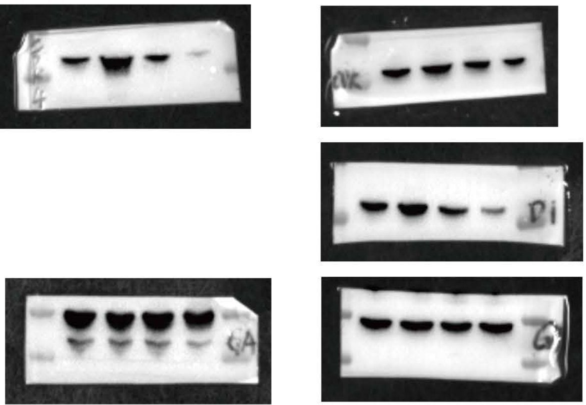

Figure4F

Figure4J

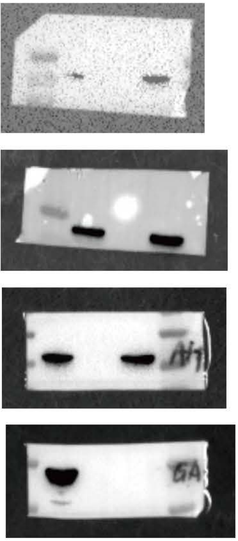

Figure4K

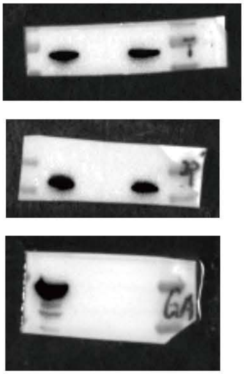

Figure4L

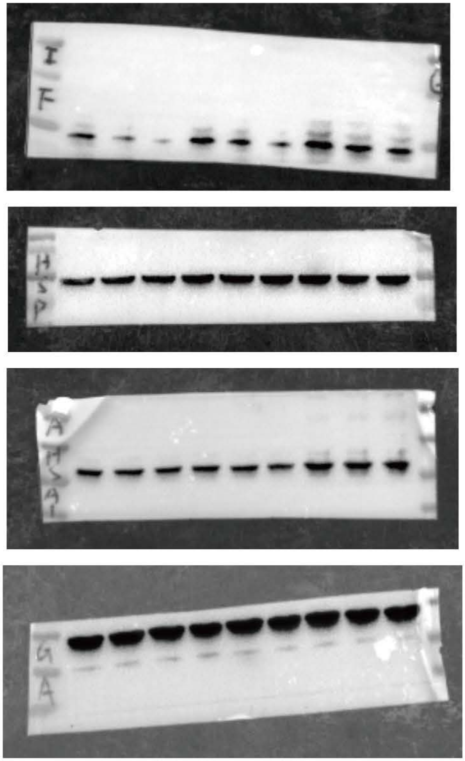

Figure4M

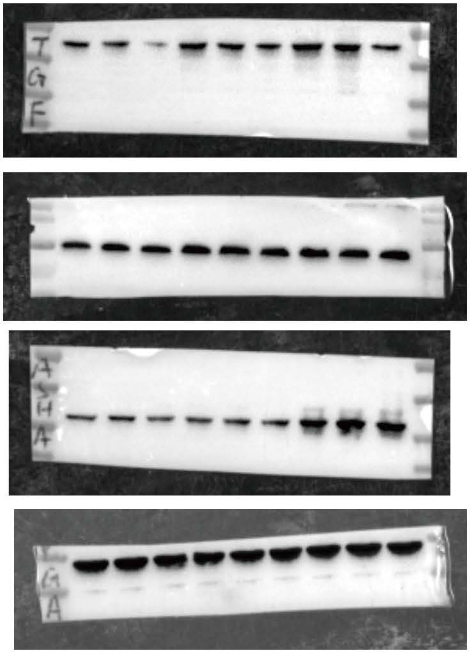

Figure5A

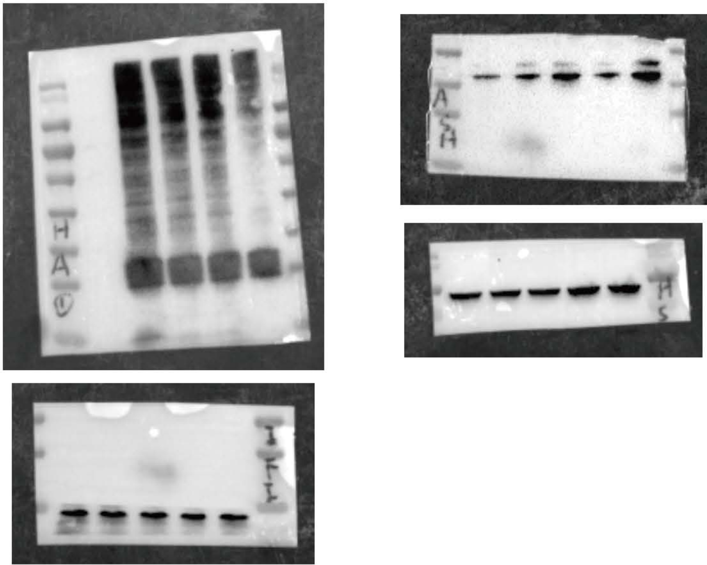

Figure5B

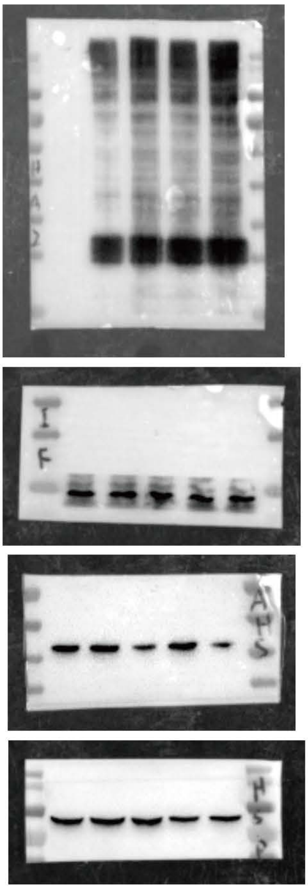

Figure5C

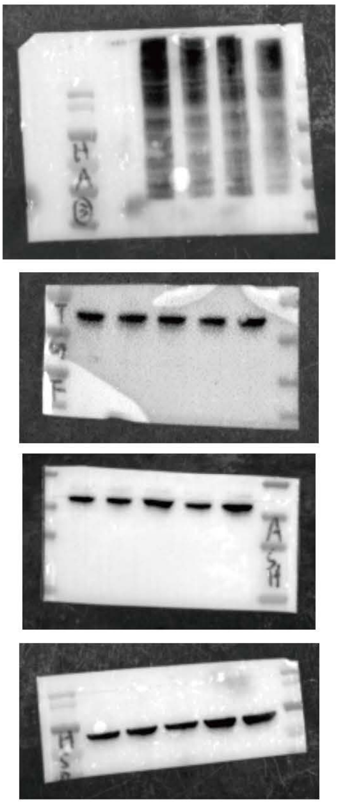

Figure5D

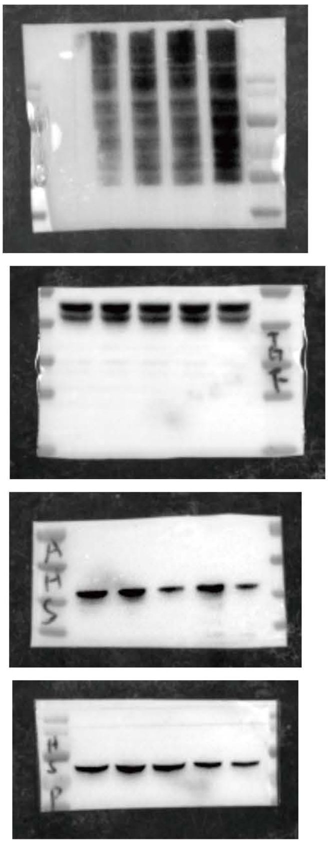

Figure5E

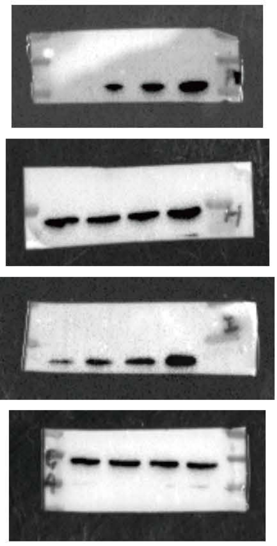

Figure5F

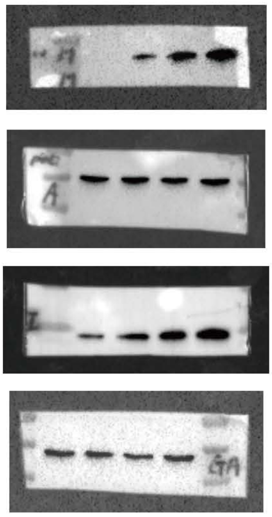

Figure5G

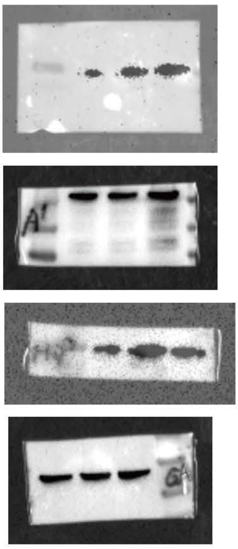

Figure5H

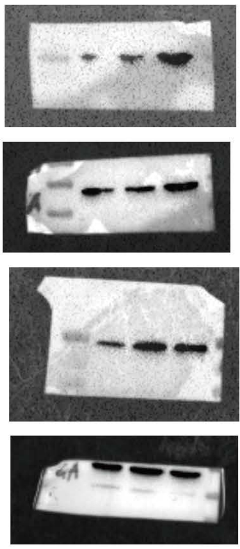

Figure5I

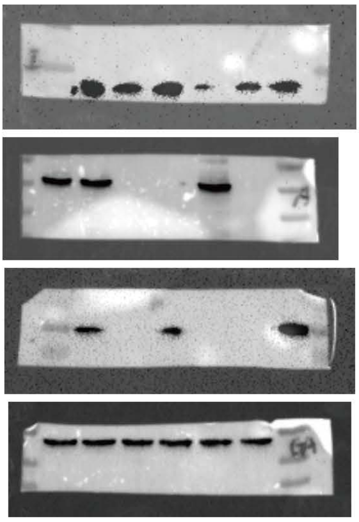

Figure5J

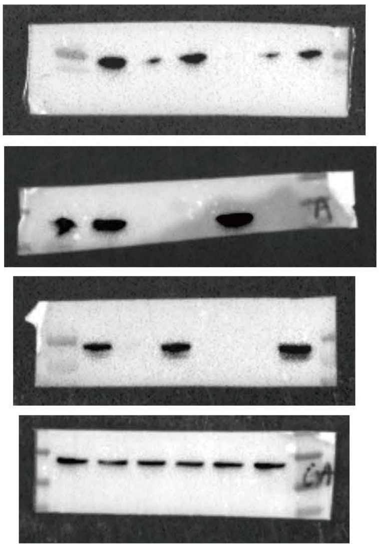

Figure5K

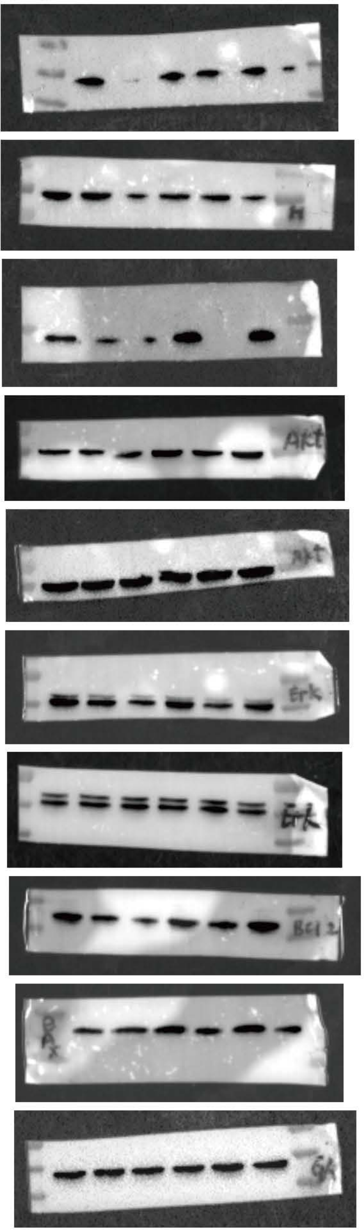

Figure5L

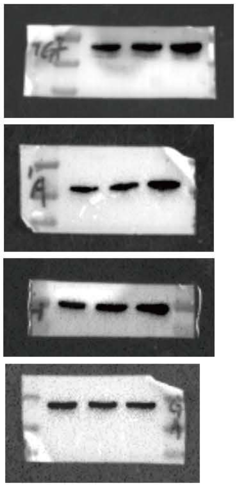

Figure5N

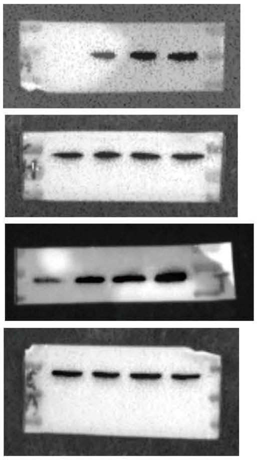

Figure5O

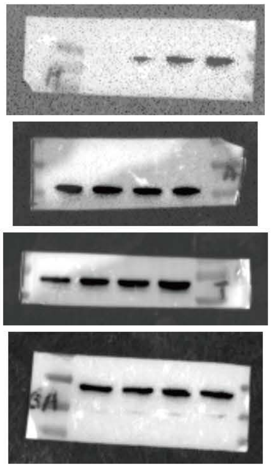

Figure5P

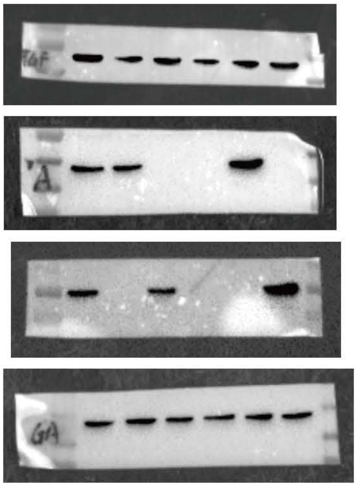

Figure5Q

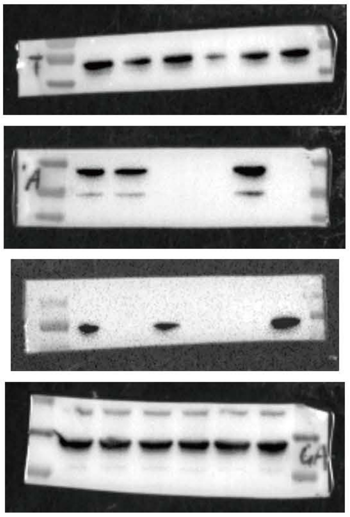

Figure5M

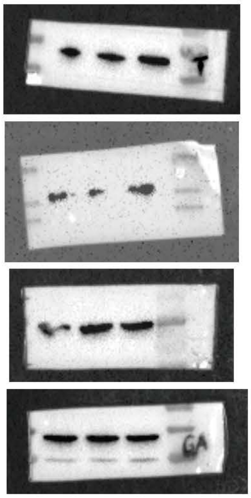

Figure6B

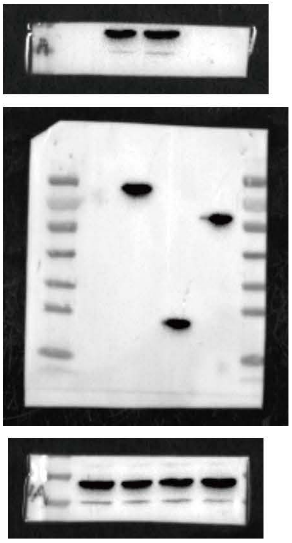

Figure6C

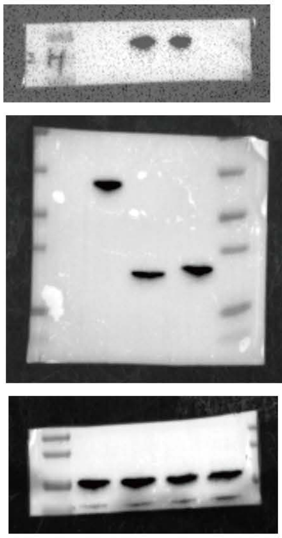

Figure6D

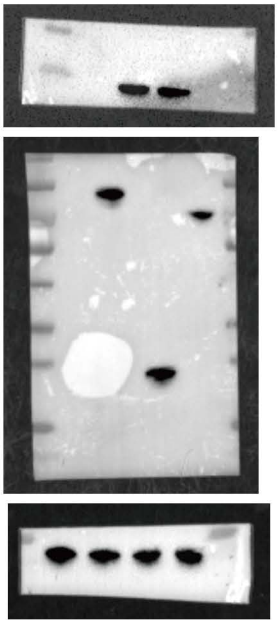

Figure6E

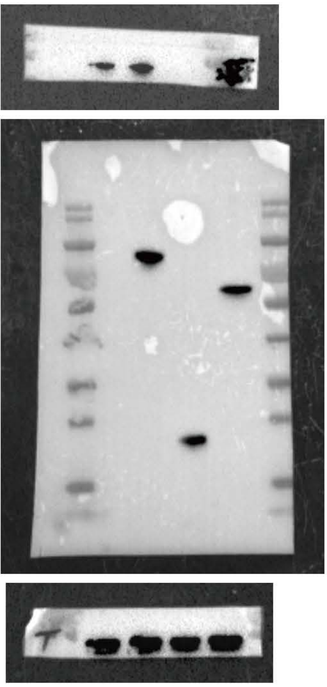

FigureS2B

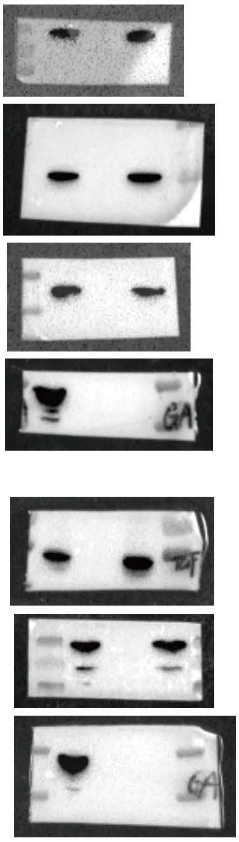

Figure6F

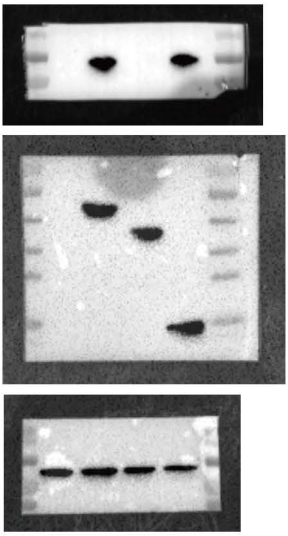

Figure7H

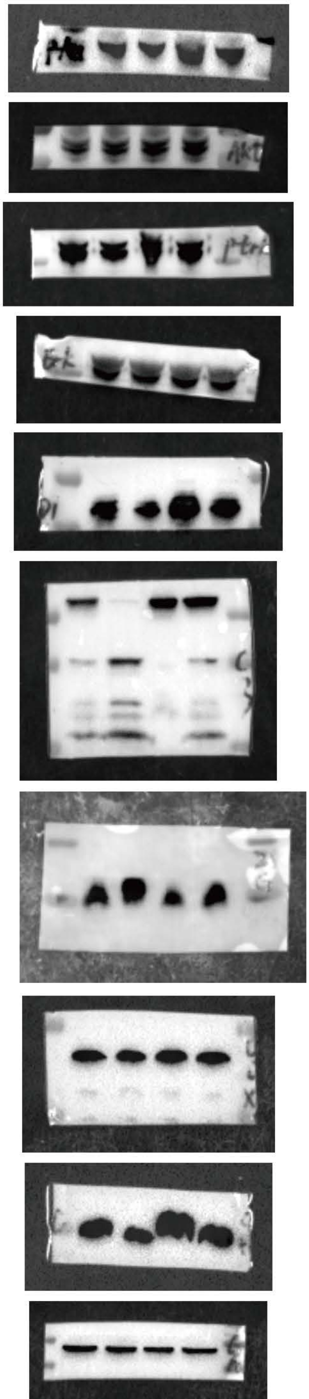

FigureS4C

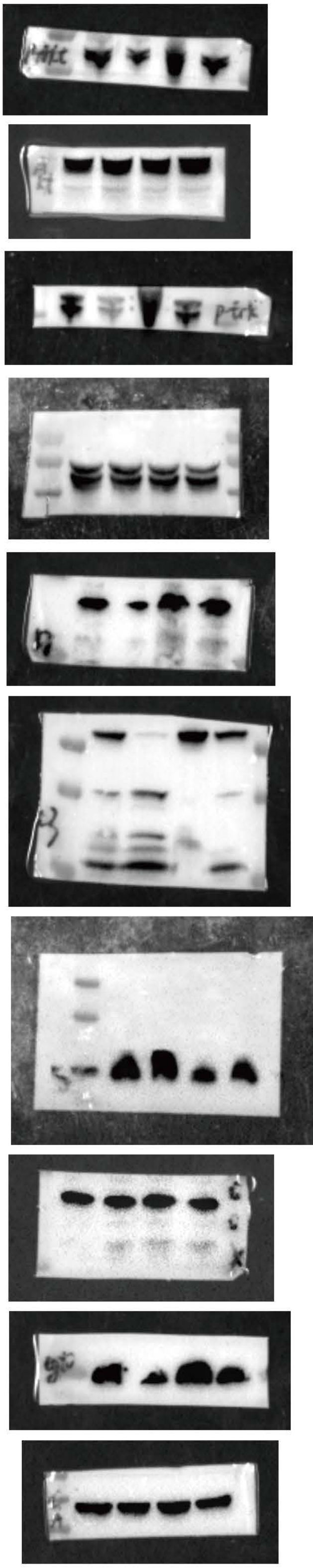

Supplement: Supplementary file 2 — Full and uncropped western blots [file 41419_2025_7650_MOESM2_ESM.pdf]
